# The glucagon-like peptide-1 receptor agonist reduces inflammation and blood-brain barrier breakdown in an astrocyte-dependent manner in experimental stroke

**DOI:** 10.1186/s12974-019-1638-6

**Published:** 2019-11-28

**Authors:** Yilong Shan, Sha Tan, Yinyao Lin, Siyuan Liao, Bingjun Zhang, Xiaodong Chen, Jihui Wang, Zhezhi Deng, Qin Zeng, Lei Zhang, Yuge Wang, Xueqiang Hu, Wei Qiu, Lisheng Peng, Zhengqi Lu

**Affiliations:** 1 Department of Rehabilitation Medicine, The Third Affiliated Hospital of Sun Yat-sen University, No. 600 Tianhe Road, Guangzhou City, China; 2 Department of Neurology, The Third Affiliated Hospital of Sun Yat-sen University, No. 600 Tianhe Road, Guangzhou City, China; 3 Department of Psychiatry, The Third Affiliated Hospital of Sun Yat-sen University, No. 600 Tianhe Road, Guangzhou City, China; 4 Department of Neurology, The Fifth Affiliated Hospital of Sun Yat-sen University, No. 52 Meihuadong Road, Zhuhai City, China

**Keywords:** Astrocyte, Exendin-4, Blood-brain barrier, Oxygen-glucose deprivation, Ischemic stroke

## Abstract

**Background:**

Preserving the integrity of the blood-brain barrier (BBB) is beneficial to avoid further brain damage after acute ischemic stroke (AIS). Astrocytes, an important component of the BBB, promote BBB breakdown in subjects with AIS by secreting inflammatory factors. The glucagon-like peptide-1 receptor (GLP-1R) agonist exendin-4 (Ex-4) protects the BBB and reduces brain inflammation from cerebral ischemia, and GLP-1R is expressed on astrocytes. However, the effect of Ex-4 on astrocytes in subjects with AIS remains unclear.

**Methods:**

In the present study, we investigated the effect of Ex-4 on astrocytes cultured under oxygen-glucose deprivation (OGD) plus reoxygenation conditions and determined whether the effect influences bEnd.3 cells. We used various methods, including permeability assays, western blotting, immunofluorescence staining, and gelatin zymography, in vitro and in vivo.

**Results:**

Ex-4 reduced OGD-induced astrocyte-derived vascular endothelial growth factor (VEGF-A), matrix metalloproteinase-9 (MMP-9), chemokine monocyte chemoattractant protein-1 (MCP-1), and chemokine C-X-C motif ligand 1 (CXCL-1). The reduction in astrocyte-derived VEGF-A and MMP-9 was related to the increased expression of tight junction proteins (TJPs) in bEnd.3 cells. Ex-4 improved neurologic deficit scores, reduced the infarct area, and ameliorated BBB breakdown as well as decreased astrocyte-derived VEGF-A, MMP-9, CXCL-1, and MCP-1 levels in ischemic brain tissues from rats subjected to middle cerebral artery occlusion. Ex-4 reduced the activation of the JAK2/STAT3 signaling pathway in astrocytes following OGD.

**Conclusion:**

Based on these findings, ischemia-induced inflammation and BBB breakdown can be improved by Ex-4 through an astrocyte-dependent manner.

## Background

The blood-brain barrier (BBB) is a physical and functional barrier between the central nervous system (CNS) and blood that maintains CNS homeostasis [1]. Astrocytes, pericytes, microglia, brain capillary endothelial cells, and even some neurons are critical components of the BBB [1, 2]. These components are activated in response to acute ischemic stroke (AIS) and then secrete inflammatory factors that directly or indirectly disrupt the integrity of the BBB. In fact, the loss of tight junction proteins (TJPs) between brain capillary endothelial cells is an indicator of BBB destruction [3, 4]. The role of astrocytes in maintaining the integrity of BBB under physiological and pathological conditions is indispensable. The integrity of in vitro BBB models comprising brain endothelial cells co-cultured with astrocytes and/or pericytes is greater than that of one comprising an endothelial cell monolayer [5]. However, under pathological conditions, such as traumatic and ischemic injury, astrocytes secrete inflammatory factors, such as vascular endothelial growth factor (VEGF-A), matrix metalloproteinases (MMPs), chemokines, and cytokines; these factors directly or indirectly aggravate brain damage and BBB disruption [6–8]. VEGF-A secreted by astrocytes directly destroys the BBB during cerebral ischemia [9]. The levels of MMPs, mainly MMP-2 and MMP-9, are increased during stroke and are related to the degradation of the basal lamina and loss of BBB integrity [6, 10]. Although microglia and neutrophils are the main sources of MMPs, astrocytes have also been reported to express MMPs under pathological conditions [11, 12]. In addition, astrocytes are a source of the chemokine monocyte chemoattractant protein-1 (MCP-1) and chemokine C-X-C motif ligand 1 (CXCL-1) [7, 13]. MCP-1 not only influences the cytokines (IL-1β, IL-6 and TNF-α) secreted by astrocytes but also plays a pivotal role in post-ischemic microglial activation [14]. On the other hand, as a neutrophil chemoattractant, a reduction in CXCL-1 levels impairs the recruitment of neutrophils after cerebral ischemia [7]. Therefore, astrocytes participate in the complex formation of inflammatory environment and the mechanism of BBB disruption following AIS.

The glucagon-like peptide-1 receptor (GLP-1R) agonist exendin-4 (Ex-4) is a long-acting analog of the endogenous insulinotropic peptide, which has been approved as a treatment for type 2 diabetes mellitus [15]. As a small molecule, this peptide diffuses across the BBB [16]. GLP-1R agonists have recently been reported to protect against ischemic stroke by reducing the degradation of TJPs between endothelial cells to maintain BBB stability in both diabetic and non-diabetic middle cerebral artery occlusion (MCAO) models and a model of traumatic brain injury [15, 16]. On the other hand, GLP-1R agonists reduce microglial activation and neutrophil infiltration in the warfarin-associated hemorrhagic transformation model, thus contributing to maintaining the integrity of the BBB [17]. However, the mechanism of the protective effect of GLP-1R agonists during AIS is still very uncertain, and no study has assessed the direct effects of GLP-1R agonists on astrocytes during AIS, although astrocytes express GLP-1Rs and contribute to both BBB disruption and inflammation activation [18]. In the current study, we investigated whether Ex-4 impacted the secretion of astrocytes and how this effect influenced the BBB integrity to provide additional evidence supporting the use of GLP-1R agonists as a treatment for AIS.

## Methods

### Cell culture

Primary cultures of cortical astrocytes were prepared from the cortices of neonatal 1-day-old C57BL/6 mice using methods described in a previous stuciefly, cortices were dissected from the brains, and the meninges were carefully removed [19]. Then, the cortices were dissociated into a cell suspension by digestion with 0.25% trypsin for 10 min at 37 °C, and the digestion was stopped with Dulbecco’s Modified Eagle’s Medium containing 4.5 g/L glucose (DMEM; Gibco, New York, NY, USA) supplemented with 10% fetal bovine serum (FBS; Gibco, New York, NY, USA), 2 mmol/L glutamine, 100 units/mL penicillin, and 100 μg/mL streptomycin. After washes with 0.01 M phosphate-buffered saline (PBS), the dissociated cells were seeded at a density of hemispheres/24-well plate or 1.5 hemispheres/6-well plate in 4.5 g/L glucose DMEM and cultured at 37 °C in a humidified atmosphere of 5% CO_2_/95% air. After 10–12 days of culture, the cells reached 60–70% confluence and were shaken (250 rpm) for 18 h to minimize microglial contamination. Adherent astrocytes in one of the 6-well plate were detached with trypsin/EDTA, and the recovered cells were plated on new 24-well plate. The cells in the new and original 24-well plates were used to confirm the purity of astrocytes using immunofluorescence staining for glial fibrillary acidic protein (GFAP). Ninety-five percent of the cultured cells were identified as astrocytes, and the adherent cells (primary astrocytes) never subjected to cell passaging in 6- or 24-well plates were used in the following experiments. The mouse brain endothelial cell line bEnd.3 was obtained from American Type Culture Collection (ATCC, Manassas, VA, USA) and cultured in 4.5 g/L glucose DMEM supplemented with 10% FBS, 2 mmol/L glutamine, 100 units/mL penicillin, and 100 μg/mL streptomycin after thawing. Then, the cell medium was replaced with new culture medium every 2–3 days, and the cells were subcultured after trypsinization at a ratio of 1:3 every 3 days until use in the experiments The bEnd.3 cells used in experiments are 3rd-5th passage.

### Exposure of astrocytes to the oxygen-glucose deprivation plus reoxygenation treatment

Control cultures were incubated with serum-free 4.5 g/L glucose DMEM at 37 °C in a 5% CO_2_/95% air atmosphere for 4 h and then refresh normal medium with (Ex4 group) or without Ex4(Medium group). Astrocytes were washed twice with PBS and incubated with glucose-free DMEM [oxygen-glucose deprivation (OGD) medium]. Then, cells were transferred to an anaerobic chamber filled with a gas mixture of 95% N_2_ and 5% CO_2_ at 37 °C for 2 h or 4 h as the OGD treatment (OGD+RO group). After OGD treatment, a germ-free glucose solution and FBS were added to the OGD medium to yield 4.5 g/L glucose DMEM supplemented with 10% FBS and cells were cultured for an additional 24 h with or without the corresponding treatment [including 10/50/100/200 nM GLP-1R activator Ex-4 (OGD+RO+Ex4 group) and/or 0.1 μM GLP-1R antagonist exendin-(9-39) (Ex[9–39] (OGD+RO+Ex4+Ex[9–39] group)] (MedChemExpress, USA) and/or 10 μM AG490 (OGD+RO+AG490 group) and 20 μM LY294002 (OGD+RO+Ex4+ LY294002 group) at 37 °C in a humidified atmosphere of 5% CO_2_/95% air.

### Culture of bEnd.3 cells with different types of astrocyte-conditioned media

After culture under normal culture conditions to 70–80% confluence, bEnd.3 cells were cultured with different types of astrocyte-conditioned medium (ACM) for another 24 h before being subjected to different experiments [20]. The grouping method in experiments involved with ACM was based on astrocyte treatment. The sources of ACM included medium from the Medium group (ACM-Medium), from the Ex-4 group (ACM-Ex4) for 24 h, from the OGD+RO group (ACM-OGD+RO), from the OGD+RO+Ex4 group (ACM-OGD+RO+Ex-4), and from astrocytes treated with OGD for 4 h plus RO for 24 h in the presence of Ex-4 and Ex(9-39) [ACM-OGD+RO+Ex-4+Ex(9-39)]. All bEnd.3 cell monolayers were pretreated with Ex(9-39) before culture with different ACMs to avoid the direct influence of Ex-4 present in the ACM on bEnd.3 cells. Cells were treated with a 10-ng/mL dose of a VEGFR2 inhibitor (VI) (R&D Systems, USA) (ACM-OGD+RO+VI) or a 50-ng/mL dose of the MMP inhibitor batimastat (MI) (MedChemExpress, USA) (ACM-OGD+RO+MI) to block VEGFR2 or MMP-9, respectively, as indicated.

### Measurement of transendothelial electric resistance

After the administration of different treatments, the transendothelial electric resistance (TEER) values of confluent bEnd3 cell monolayers were measured using a voltage/ohm meter (Millipore, Billerica, MA, USA). The TEER of blank inserts was subtracted from the measured TEER of each group to reflect that of cell monolayers themselves. The values are expressed as Ω cm^2^.

### Paracellular permeability assay

Briefly, bEnd.3 cells were seeded onto polycarbonate 12-well transwell inserts with a 0.4-μm mean pore size and a 0.33-cm^2^ surface area (Corning, USA) at a density of 4 × 10^4^ cells/cm^2^, and the growth medium was refreshed every other day. The seeded cells were allowed to grow at 37 °C in a 5% CO_2_/95% air atmosphere until they reached 70–80% confluence, which was confirmed using phase contrast microscopy. Then, cells were cultured with different ACMs for 24 h. After two washes with PBS, the media in the transwell inserts were replaced with media supplemented with 500 μL of 1 mg/mL sodium fluorescein (NaF), the lower chamber was filled with 1000 μL of normal media, and cells cultured for 1 h at 37 °C under normoxic conditions. Relative fluorescence passing through the chamber was measured as follows: 100 μL of medium in the lower chamber was assayed in triplicate in black 96-well plates (Corning, USA). The fluorescence intensity was measured using an EnSpire Manager (PerkinElmer, USA) multimode plate reader at an excitation wavelength of 460 nm and an emission wavelength of 515 nm [11]. NaF permeability (μg/cm^2^) = total NaF quantity in the lower chamber/the surface area of insert (0.33 cm^2^).

### Western blotting

Confluent astrocytes or bEnd.3 cells at 70–80% confluence cultured in 6-well plates were exposed to the corresponding culture conditions. After different treatments, total protein was collected by lysing the cells with RIPA buffer (Beyotime Biotechnology, China). The protein content in each well was determined using a BCA protein assay kit (Beyotime Biotechnology, China). Lysates containing equal amounts of total protein were separated on polyacrylamide gels (6% or 10%) and then transferred onto polyvinylidene fluoride (PVDF) membranes (Millipore, USA). Membranes were blocked with 3% bovine serum albumin (BSA) for 1 h at room temperature (RT) and incubated with the following primary antibodies: GAPDH (Beyotime Biotechnology, China); claudin-5, occludin, and zonula occludens-1 (ZO-1) (Invitrogen, USA); endothelial nitric oxide synthase (eNOS) and VEGF-A (Abcam, UK); and phosphorylated phosphatidylinositol 3-kinase (p-PI3K), total PI3K, p-protein kinase B (p-Akt), total Akt, p-phospholipase Cγ (p-PLCγ), total PLCγ, p-protein kinase Cα (p-PKCα), total PKCα, p-Janus kinase 2 (p-JAK2), total JAK2, p-signal transducer and activator of transcription 3 (p-STAT3), and total STAT3 [all from Cell Signaling Technology (CST), USA] at 4 °C overnight. Membranes were washed with Tris-buffered saline containing Tween 20 (TBST) (10 mM Tris, pH 8.0, 150 mM NaCl, and 0.1% Tween 20) before the addition of horseradish peroxidase-conjugated secondary antibodies (CST, USA) for 1 h at RT. After three washes with TBST, the signals were visualized with a Tanon Imaging System (China), and the density of each band was quantified using ImageJ software (National Institutes of Health, USA).

### Immunofluorescence staining

Twenty-four hours after MCAO, the rats were anesthetized and first perfused with saline followed by fixation with a 4% buffered paraformaldehyde solution. The brains were removed and postfixed with 4% paraformaldehyde; the paraformaldehyde was then removed and replaced with a 30% sucrose solution overnight. Then, 8-μm coronal sections were obtained using a cryostat. Sections were incubated with 0.3% Triton X-100 at RT. After three washes with PBS three, brain slices were blocked with PBS containing 5% BSA for 1 h at RT. In vitro cell specimens, after different treatment, confluent bEnd.3 cells and astrocytes growing on collagen-coated coverslips were subjected to the aforementioned treatments and washed three times with PBS before 4% paraformaldehyde fixation. Fixed cells were permeabilized with 0.3% Triton X-100 for 15 min and blocked with 10% goat serum in PBS for 1 h at RT. After that, sections or fixed cells were incubated at 4 °C overnight with the following primary antibodies: GFAP (CST); VEGF-A (Abcam); MMP9 (Invitrogen); claudin-5 and and ZO-1 (Invitrogen); occludin (Abcam); and GLP-1R (Bioss). After three washes with PBS, an Alexa Fluor 488- or 555-labeled secondary antibody (Invitrogen) was added and incubated for 1 h at RT. Tissue sections or cells specimens were washed twice with PBS and rinsed with a DAPI solution. Tissue sections were covered with a coverslip that contained a drop of antifade mounting medium. Images of both brain slices and cells on coverslips were acquired with a fluorescence microscope or with an LSM800 confocal microscope.

### Immunohistochemistry

Twenty-four hours after MCAO, the rats were anesthetized and first perfused with saline followed by fixation with ice-cold 4% paraformaldehyde. Brain tissues were removed and fixed overnight in 4% paraformaldehyde at 4 °C and then immersed in 30% sucrose. The tissues were cut to 2-mm-thick coronal sections and embedded in paraffin and further sliced at a thickness of 5 μm. The slides were deparaffinized, sequentially rehydrated in graded alcohol, and then immersed in PBS (pH 7.4). The slides were then microwaved for 2 min in antigen-unmasking solution, cooled, and washed three times for 2 min in PBS. Sections were immersed for 25 min in 3% hydrogen peroxide in distilled water to eliminate endogenous peroxidase activity, then blocked in immunohistochemical grade 1% bovine serum albumin in PBS for 1 h and diluted goat serum for 30 min to reduce nonspecific staining. Sections were incubated overnight with primary antibodies anti-CXCL1 (Invitrogen) or anti-MCP1 (Abcam). Peroxidase-conjugated anti-rabbit IgG (BD Biosciences) was used as the secondary antibody and was incubated for 30 min at 37 °C. Antibodies were detected using the DAB kit (Beyotime Biotechnology, China) following the manufacturer’s instructions. Finally, the slides were observed using a light microscope. The positive cells were expressed as the number of immunopositive cells/mm^2^.

### Lactate dehydrogenase assay

The viability of bEnd.3 cells was evaluated by quantifying plasma membrane damage, which resulted in the release of LDH. The level of Lactate dehydrogenase (LDH) released in the cell culture supernatant was detected using an LDH cytotoxicity assay detection kit (Beyotime, China) according to the manufacturer’s instructions.

### Enzyme-linked immunosorbent assay

The cell culture supernatants of astrocytes subjected to different treatments were harvested and assayed with Enzyme-linked immunosorbent assays (ELISAs) to determine the levels of tumor necrosis factor-α (TNF-α), interleukin (IL)-1β, IL-6, transforming growth factor-β (TGF-β), CXCL-1, VEGF-A (R&D Systems, USA), and MCP-1 (Sigma-Aldrich, USA) according to the manufacturer’s protocols. The results were calculated by measuring the absorbance at a wavelength of 450 nm using a multiplate reader (Bio-Tek, Winooski, VT, USA). The experiments were performed in triplicate.

### Real-time quantitative PCR

Total RNA was extracted from astrocytes using TRIzol (Invitrogen, USA) according to the manufacturer’s instructions. The cDNA templates were synthesized from total RNA using a Transcriptor First Strand cDNA Synthesis kit (TaKaRa Biotechnology). TaqMan primers and probes used for testing were obtained from TaKaRa Biotechnology, Japan. GAPDH was used as an endogenous reference. Quantitative PCR using SYBR Green II (TaKaRa Biotechnology) was performed with an ABI PRISM 7900 Sequence Detector system (Applied Biosystems). Target gene expression was normalized to GAPDH expression, and the values were calculated relative to control values using the ΔΔCT method. The following PCR primers were used (5′ to 3′): IL-1β-F, GACCTGTTCTTTGAGGCTGACA and IL-1β-R, CTCATCTGGACAGCCCAAGTC; IL-6-F, TAGTCCTTCCTACCCCAACTTCC and IL-6-R, TTGGTCCTTAGCCACTCCTTC; TGF-β-F, CATTGCTGTCCCGTGCAGA and TGF-β-R, AGGTAACGCCAGGAATTGTTGCTA; TNF-α-F, TTCCAATGGGCTTTCGGAAC and TNF-α-R, AGGTAACGCCAGGAATTGTTGCTA; VEGF-A-F, TCCTGCAGCATAGCAGATGTGA and VEGF-A-R, CCAGGATTTAAACCGGGATTTC; MMP-2-F, TCCCGAGATCTGCAAGCAAG and MMP-2-R, AGAATGTGGCCACCAGCAAG; MMP-9-F, GGGAACGTATCTGGAAATTCGAC and MMP-9-R, CCGGTTGTGGAAACTCACAC; MCP-1-F, TGTCTCAGCCAGATGCAGTTAAT and MCP-1-R, CCGACTCATTGGGATCATCTT; CXCL-1-F, CAATGAGCTGCGCTGTCAGT and CXCL-1-R, TTGAAGTGAATCCCTGCCACT; and GAPDH-F, GGCACAGTCAAGGCTGAGAATG and GAPDH-R, ATGGTGGTGAAGACGCCAGTA.

### Gelatin zymography

The same number of astrocytes was seeded in each well of a 6-well plate and grown to confluence. After the administration of different treatments (all in serum-free media for 24 h), equal amounts of proteins from ACM were collected and concentrated using centrifugal filters. After electrophoresis on an 8% SDS-PAGE gel containing 1 mg/mL gelatin, gels were washed with 2.5% Triton X-100 for 30 min. Then, the gel was incubated in a developing buffer (20 mM Tris-HCl, pH 7.8, 1% Triton X-100, 10 mM CaCl_2_, 5 μM ZnCl_2_) for 24 h at 37 °C. Thereafter, the gel was stained with 1% Coomassie Brilliant Blue R-250. The gelatinolytic activities of MMP-9 were detected as transparent bands.

### Experimental ischemic stroke model and treatment

All animal experiments were approved by the Ethics Committee of Sun Yat-sen University. Male SD rats weighing 220–250 g were randomly assigned to different groups using a random number table. The rats were anesthetized with 2% pentobarbital (50 mg/kg, i.p.). The MCAO model of ischemic stroke was induced by a left side occlusion of the middle cerebral artery with silicone-coated sutures as previously described [21]. After 90 min of occlusion, the filament was removed to allow reperfusion. The rats in the treatment group were administered Ex-4 and/or 3000 μg/kg Ex[(9-39)] (in saline at a total volume of 1 mL) or saline by intraperitoneal injections immediately after reperfusion. The doses of Ex-4 were 50, 150, and 300 μg/kg to determine the effective dose that suitable for subsequent experiments.

### Neurological deficit score

Neurological function was evaluated at the time rats recovered from anesthesia (approximately 3 h) and 24 h after MCAO. The deficits were scored on a modified scoring system based on that developed by Longa et al. [21] as follows: 0, no neural defective symptoms; 1, rats cannot stretch the contralateral front paws; 2, rats circle to the contralateral side while crawling; 3, rats tumble to the contralateral side while crawling; 4, rats cannot walk independently or lose consciousness or death. A first score of 2–3 points was considered a successful model, and these rats were included in the experimental group according to the treatment.

### Quantification of infarct area

The brain was removed and immediately sliced into 1-mm-thick sections. The slices were then stained with a 2% solution of 2,3,5-triphenyltetrazolium chloride (TTC) at 37 °C for 30 min. The infarcted area of each brain slice was measured by ImageJ analysis software, and the percentage hemisphere lesion area was calculated as follows: {[total infarct area-(left hemisphere area-right hemisphere area)]/right hemisphere area} × 100% [22].

### Evans blue dye extravasation

Leakage of Evans blue (EB, Sigma-Aldrich, USA) dye in the ischemic brain tissue, which is indicative of BBB disruption, was evaluated after the 90-min MCAO procedure (with or without Ex-4/Ex(9-39) treatment) or after the sham operation (sham group). EB (2%) in normal saline (6 mL/kg) was intravenously injected into the animals and allowed to circulate for 3 h before sacrifice. After sacrifice, every 100 mg of the collected ischemic-side brain tissue was added with 1 mL of 50% trichloroacetic acid solution to extract the EB. After homogenization of the brain tissue, the mixture was centrifuged at 15000*g* for 15 min, and the supernatant was harvested. The supernatant was diluted 4-fold with ethanol, and the mixture was allowed to stand at room temperature for 30 min. The amount of EB in the ischemic tissue was quantified at 610 nm by a spectrophotometer according to a standard curve of optical density (OD) obtained according to different concentrations of EB.

### Statistical analysis

All data are presented as the mean ± SD. The data were analyzed using a two-tailed *t* test or one-way analysis of variance (ANOVA) followed by the post hoc Student-Newman-Keuls (SNK) *t* test for multiple comparisons. Differences were considered significant at *P* < 0.05. Statistical analyses were performed using SPSS 18.0 software.

## Results

### OGD-treated astrocytes increased the permeability of confluent endothelial cells

**Fig. 1:**
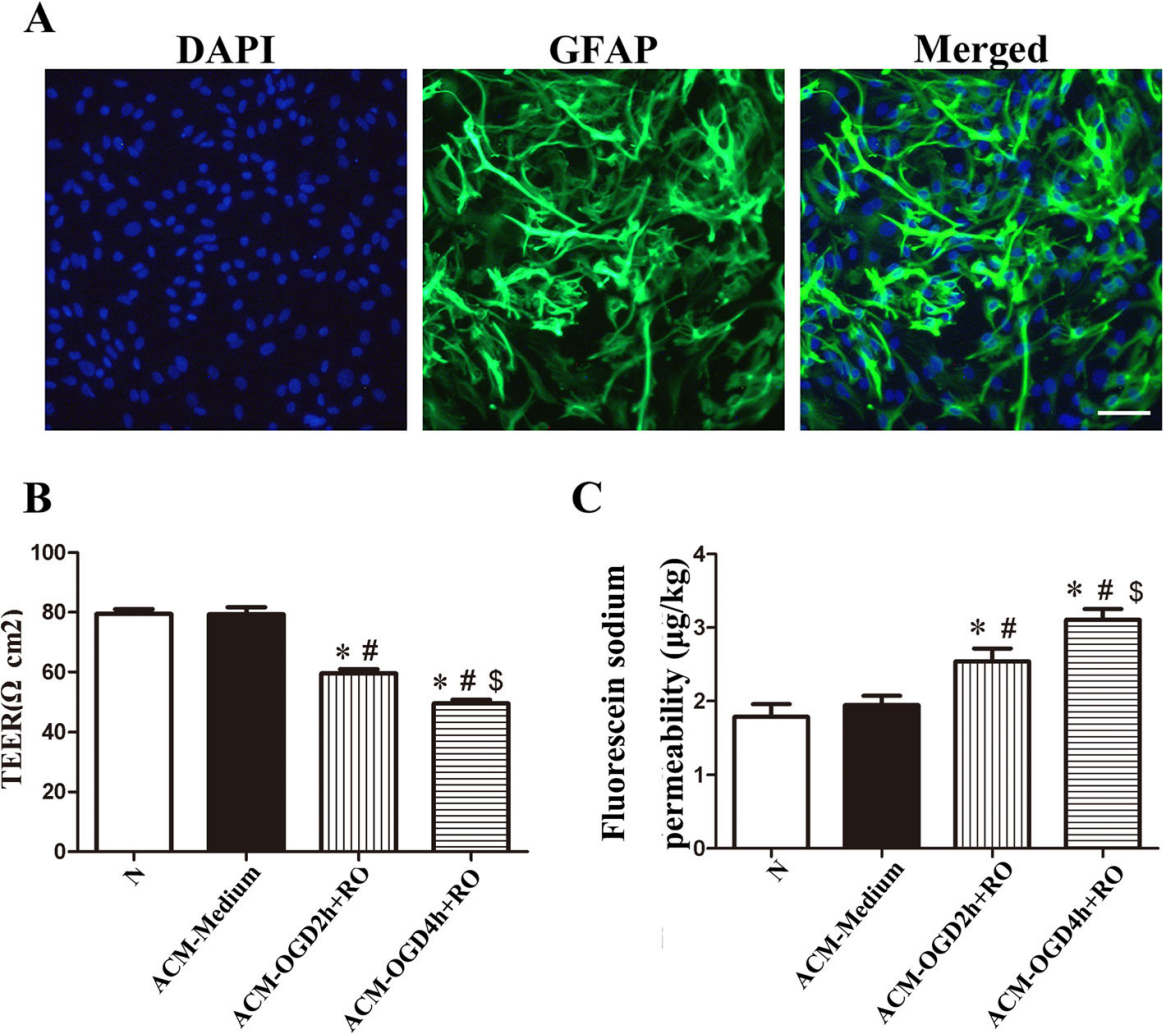
Astrocytes exposed to OGD plus RO increased the permeability of the bEnd.3 monolayer. **a** GFAP (green) was expressed in more than 95% of cultured cells, and the nuclei were counterstained with DAPI (blue). Scale bar, 100 μm. **b** The TEER value of bEnd.3 monolayer cultured with different ACMs for 24 h was assessed. **c** The ability of sodium fluorescein (NaF) to cross the bEnd.3 monolayer cultured with different ACMs for 24 h was assessed (*n* = 7). N, cultured with normal 10% FBS DMEM; ACM-Medium, cultured with ACM from untreated astrocytes; ACM-OGD 2 h+RO, cultured with ACM from astrocytes exposed to OGD for 2 h plus RO for 24 h; ACM-OGD 4 h+RO, cultured with ACM from astrocytes treated with OGD for 4 h plus RO for 24 h. *P < 0.05 compared with the N group; ^#^*P* < 0.05 compared with the ACM-Medium group; and ^$^*P* < 0.05 compared with the ACM-OGD 2 h+RO group, ANOVA plus SNK test (**b**, **c**)

Cultured cells were identified as astrocytes because they were GFAP-positive (Fig. 1a). After astrocyte exposure to normoxia or OGD, the normal medium of bEnd.3 cells was replaced with different ACMs. ACM from normally cultured astrocytes did not change the TEER of confluent bEnd.3 cells (ACM-Medium vs. *N*, 79.20 ± 2.40 vs. 79.43 ± 1.59 Ω cm^2^). After OGD treatment followed by 24 h of RO, ACM from OGD+RO-treated astrocytes significantly decreased the TEER of confluent bEnd.3 cells (ACM-Medium, 79.20 ± 2.40; ACM-OGD2h+RO, 59.53 ± 1.41, *P* < 0.05; ACM-OGD4h+RO, 49.43 ± 1.29 Ω cm^2^, *P* < 0.05), and the damage increased as the duration of OGD increased (Fig. 1b). The tendency of NaF permeability in bEnd.3 cells caused by different ACMs was the opposite to that of TEER of bEnd.3 cells (NaF permeability: ACM-Medium, 1.94 ± 0.13; ACM-OGD2h+RO, 2.54 ± 0.18, *P* < 0.05; ACM-OGD4h+RO, 3.11 ± 0.14 μg/cm^2^, *P* < 0.05) (Fig. 1c). Based on these findings, OGD-treated astrocytes may have increased the permeability of endothelial cells by secreting detrimental factors. Because the medium from OGD4h+RO-treated astrocytes influenced the permeability of bEnd.3 cells to a greater extent than the medium from OGD2h+RO-treated astrocytes, the OGD treatment was performed for 4 h in subsequent experiments.

### ACM containing Ex-4 protected the integrity of the endothelial cell barrier by reducing TJP degradation

**Fig. 2:**
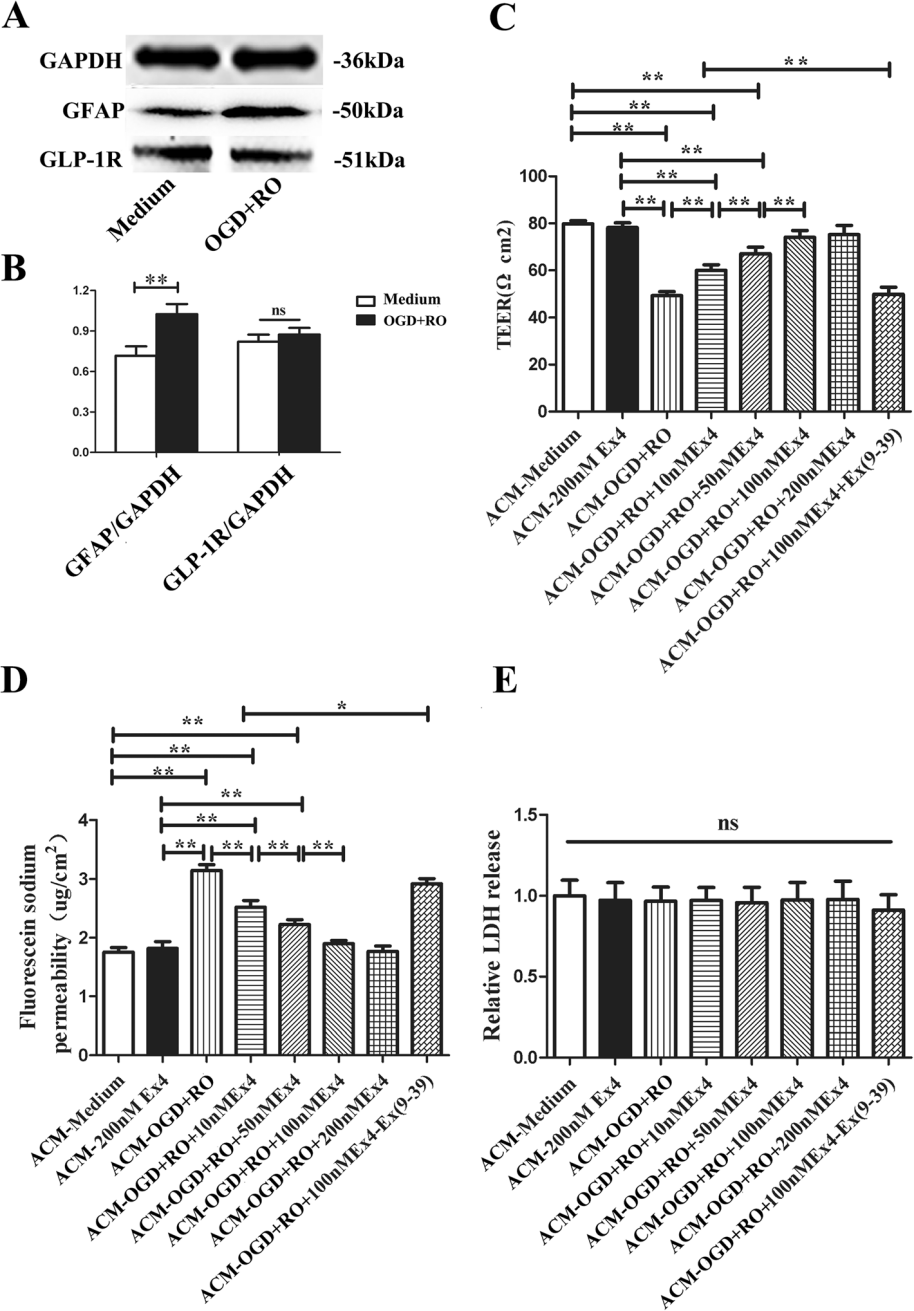
ACM from astrocytes treated with Ex-4 reduced the OGD**+**RO-induced increase in bEnd.3 cell permeability. **a** Representative western blots for GFAP and GLP-1R in astrocytes, along with the loading control GAPDH. **b** Gray values for GFAP and GLP-1R were normalized to GAPDH. The data are presented as the mean ± SD (*n* = 6). **c** The TEER value of bEnd.3 monolayer cultured with different ACMs for 24 h was assessed. **d** The ability of NaF to cross the bEnd.3 monolayer cultured with different ACMs for 24 h was assessed. **e** LDH release of bEnd.3 monolayer cultured with different ACMs for 24 h was assessed. ACM-Medium, cultured with ACM from untreated astrocytes; ACM-200 nM Ex4, cultured with ACM from astrocytes treated with 200 nM Ex-4 for 24 h; ACM-OGD+RO, cultured with ACM from astrocytes treated with OGD for 4 h plus RO for 24 h; ACM-OGD+RO +Ex-4, cultured with ACM from astrocytes treated with OGD for 4 h plus RO for 24 h in the presence of different Ex-4 concentrations. All bEnd.3 cell monolayers were pretreated with Ex(9-39) before culture with different ACMs. *P < 0.05 and **P < 0.01, ANOVA plus SNK test (**c**–**e**) or Student’s *t* test (**b**)

**Fig. 3:**
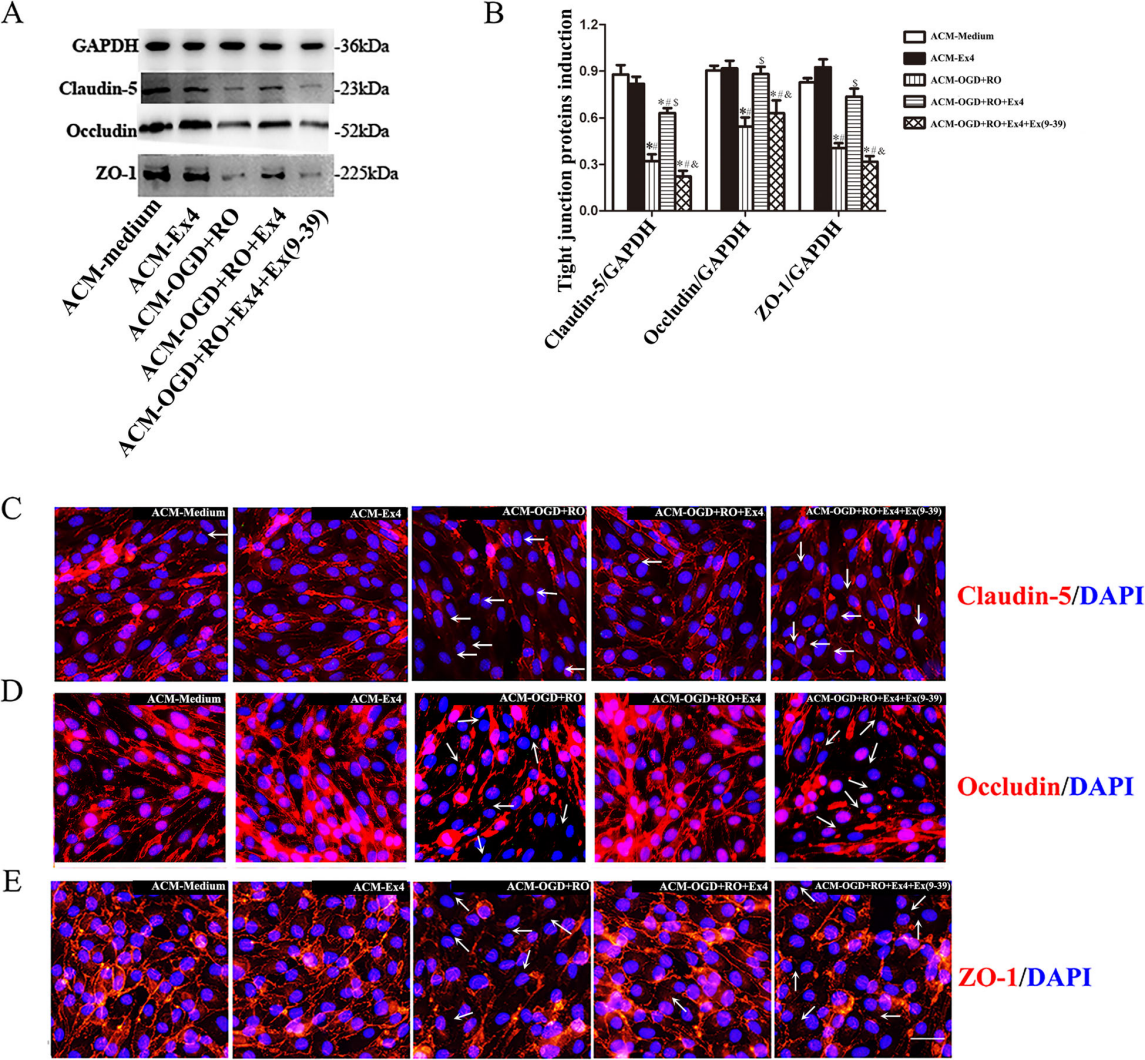
ACM from astrocytes treated with Ex-4 ameliorated the OGD**+**RO-induced destruction of TJPs in bEnd.3 cells. **a** Representative western blots showing the levels of TJPs, including claudin-5, occludin, and ZO-1 in bEnd.3 cells, along with the loading control GAPDH. **b** TJP expression was normalized to GAPDH. Quantitative results are presented as the mean ± SD (*n* = 6). **c**–**e** Immunofluorescence staining for the TJPs (claudin5, occludin, and ZO-1) (red) and nuclear staining with DAPI (blue) in bEnd.3 monolayers cultured with different ACMs (*n* = 6). The arrows indicated decreased levels of TJPs between bEnd.3 cells. Scale bar = 50 μm. ACM-Medium, cultured with ACM from untreated astrocytes; ACM-Ex4, cultured with ACM from astrocytes treated with Ex-4 for 24 h; ACM-OGD+RO, cultured with ACM from astrocytes treated with OGD for 4 h plus RO for 24 h; ACM-OGD+RO+Ex4, cultured with ACM from astrocytes treated with OGD for 4 h plus RO for 4 h in the presence of 100 nM Ex-4; ACM-OGD+RO+Ex4+Ex(9-39), cultured with ACM from astrocytes treated with OGD for 4 h plus RO for 24 h in the presence of both Ex4 and Ex(9-39); all bEnd.3 cell monolayers were pretreated with Ex(9-39) before culture with different ACMs. *P < 0.05 compared with the ACM-Medium group; ^#^*P* < 0.05 compared with the ACM-Ex4 group; ^$^*P* < 0.05 compared with the ACM-OGD+RO group; and ^&^*P* < 0.05 compared with the ACM-OGD+RO+Ex4group, ANOVA plus SNK test (**b**)

Next, we measured the levels of GFAP and GLP-1R on astrocytes using western blotting. GLP-1R levels were not significantly altered, while GFAP showed increased expression in response to OGD+RO induction (Fig. 2a and b). After exposure to normoxia or OGD, astrocytes were incubated with 10, 50, 100, or 200 nM Ex-4 for 24 h, and then, different ACMs were added to the bEnd.3 cell cultures for another 24 h; the bEnd.3 cells were all simultaneously cultured with Ex(9-39) to avoid a possible direct effect of Ex-4. As shown in Fig. 2c and d, when the cells were cultured with ACM from astrocytes treated with OGD+RO in the presence of 10 nM Ex-4 (ACM-OGD+RO+10nMEx4 group), the TEER of bEnd.3 cells significantly increased, while the NaF permeability significantly decreased compared with cells in ACM-OGD+RO group (TEER 60.06 ± 2.32 vs. 49.39 ± 1.69 Ω·cm^2^, *P* < 0.05; 2.78 ± 0.07 vs. 3.20 ± 0.15 μg/cm^2^, *P* < 0.01). When the Ex-4 concentration was increased gradually to 100 nM, the TEER significantly increased, and the NaF permeability significantly decreased (all *P* < 0.05); in contrast, 200 nM Ex-4 treatment (ACM-OGD+RO+200nMEx4 group) did not significantly affect either the TEER or NaF permeability of bEnd.3 cells compared with the ACM-OGD+RO+100nMEx4 group (*P* = 0.77, *P* = 0.64, respectively). However, exposure to Ex-4 alone did not change the impact of ACM on either the TEER or NaF permeability of bEnd.3 cells (ACM-Medium vs. ACM-Ex4, TEER, 79.78 ± 1.36 vs. 78.22 ± 1.93 Ω·cm^2^, *P* = 0.44; NaF permeability, 2.01 ± 0.14 vs. 1.86 ± 0.05 μg/cm^2^, *P* = 0.35) and the effect of Ex-4 on OGD-treated astrocyte could be blocked by GLP-1R antagonist Ex(9-39). Overall, Ex-4 reduced the ability of OGD-treated astrocytes to destroy the integrity of endothelial cells in a dose-dependent manner. Next, we measured LDH release from bEnd.3 cells cultured with different ACMs to determine whether the changes in permeability and TEER were related to cell injury, but no significant differences were observed among any of the groups (Fig. 2e). Therefore, we next examined the levels TJPs in bEnd.3 cells cultured with different ACMs. The levels of important TJPs of bEnd.3 cells (claudin-5, occludin and ZO-1) were significantly reduced in the ACM-OGD+RO group compared with the ACM-Medium group, but the levels were restored in the ACM-OGD+RO+Ex-4 group (ACM-Medium vs. ACM-OGD+RO, *P* < 0.05; ACM-OGD+RO vs. ACM-OGD+RO+Ex-4, *P* < 0.05) (Fig. 3a and b). Notably, the effect of Ex-4 on astrocytes was abolished by Ex(9-39), which was evident as the TJP levels decreased in bEnd.3 of the ACM ACM-OGD+RO+Ex4+Ex(9-39) group [ACM-OGD+RO+Ex4 vs. ACM-OGD+RO+Ex4+Ex(9-39), *P* < 0.05] (Fig. 3a and b). The results were confirmed using single-label immunofluorescence staining; the arrows indicate the downregulation of TJPs between bEnd.3 cells (Fig. 3c, d, and e). From the perspective of astrocytes, the Ex-4-mediated protection of the BBB was related to the conservation of TJPs.

### Ex-4 reduced OGD+RO-induced astrocyte-derived VEGF-A, MMP-9, MCP-1, and CXCL-1 secretion

**Fig. 4:**
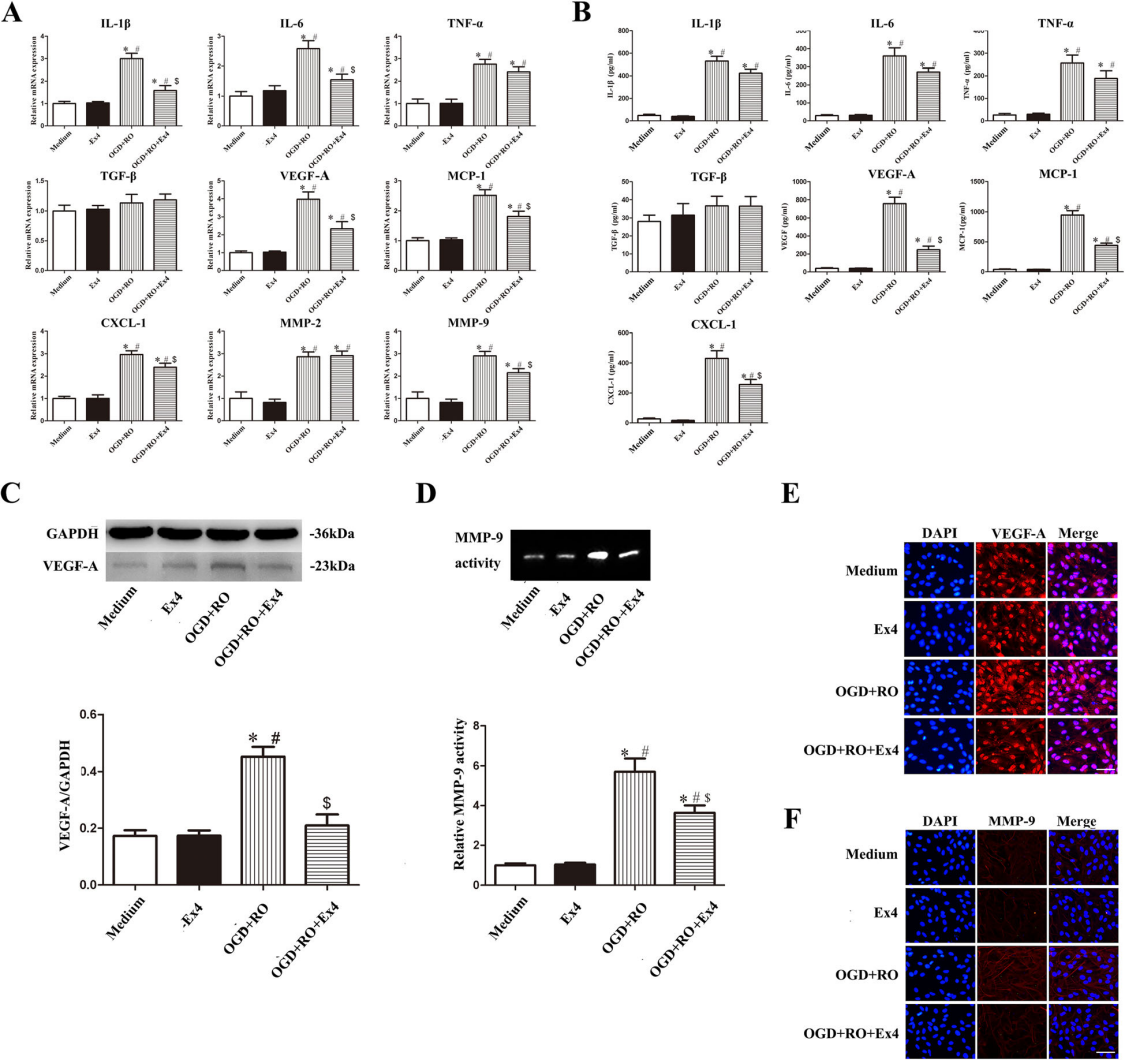
Ex-4 reduced the expression of VEGF-A, MMP-9, CXCL-1, and MCP-1 in OGD+RO-treated astrocytes in vitro. **a** The expression of the IL-1β, IL-6, TNF-α, TGF-β, VEGF-A, MCP-1, CXCL-1, MMP-2, and MMP-9 mRNAs in astrocytes subjected to different treatments in vitro was measured using qRT-PCR; GAPDH was used as the control (*n* = 6). **b** Levels of the IL-1β, IL-6, TNF-α, TGF-β, VEGF-A, MCP-1, and CXCL-1 proteins secreted by astrocytes exposed to different treatments were measured using ELISAs (*n* = 6). **c** Representative western blots showing VEGF-A expression in astrocytes administered different treatments, and the VEGF-A intensity was normalized to GAPDH (*n* = 6). **d** The levels of MMP-9 secreted from astrocytes exposed to different treatments were measured using gelatin zymography, and the relative intensity was compared with the Medium group (*n* = 6). **e**, **f** Representative images of immunofluorescence staining for VEGF-A (**e**) and MMP-9 (**f**) (red) in astrocytes; nuclei were labeled with DAPI (blue) (*n* = 5). Scale bar = 100 μm. Medium, cultured with normal medium; Ex4, treated with Ex4 for 24 h; OGD+RO, treated with OGD for 4 h plus RO for 24 h; OGD+RO+Ex4, treated with OGD for 4 h plus RO for 4 h in the presence of Ex-4. *P < 0.01 compared with the Medium group; ^#^*P* < 0.01 compared with the Ex4 group; ^$^*P* < 0.01 compared with the OGD+RO+Ex4 group, ANOVA plus SNK test (**a**–**d**)

Astrocytes activated by ischemia and anoxia secrete many pro- and anti-inflammatory factors, such as IL-1β, IL-6, TNF-α, TGF-β, VEGF-A, MCP-1 CXCL-1, MMP-2, and MMP-9, which influence BBB integrity. Next, we examined whether Ex-4 regulates the levels of these factors on astrocytes. The effects of OGD and Ex-4 on the levels of the IL-1β, IL-6, TNF-α, TGF-β, VEGF-A, MCP-1 CXCL-1, MMP-2, and MMP-9 mRNAs were assessed using real-time quantitative PCR (qRT-PCR). TGF-β was the only factor whose expression was not influenced by OGD and Ex-4 at the mRNA level. OGD significantly increased the levels of the IL-1β, IL-6, TNF-α, VEGF-A, MCP-1, CXCL-1, MMP-2, and MMP-9 mRNAs in astrocytes (3.00-, 2.58-, 2.75-, 3.98-, 2.51, 2.97, 2.87-, and 2.91-fold, respectively, compared with the normal cultured astrocytes, *P* < 0.05) (Fig. 4a). However, Ex-4 treatment decreased the OGD+RO-induced increases in the levels of the IL-1β, IL-6, VEGF-A, MCP-1, CXCL-1, and MMP-9 mRNAs (decreased to 53.67%, 60.08%, 58.94%, 72.11%, 80.47%, and 73.88%, respectively, *P* < 0.05) (Fig. 4a), but did not significantly alter the levels of the TNF-α and MMP-2 mRNAs. ELISAs were used to determine the protein levels. Ex-4 decreased only the levels of the VEGF-A, MCP-1, and CXCL-1 protein that were increased by OGD+RO in astrocyte cultures (OGD+RO vs. OGD+RO+Ex4 group, 247.17 ± 41.12 vs. 759.43 ± 68.92, 441.70 ± 36.28 vs. 946.74 ± 74.06, 256.74 ± 33.69 vs. 431.1 ± 50.43 pg/mL, respectively, *P* < 0.01) (Fig. 4b). The data from western blot analyses were consistent with the ELISA results, as Ex-4 decreased the levels of the VEGF-A protein under the OGD+RO condition (*P* < 0.05) (Fig. 4c). Based on the gelatin zymography data, the activity of astrocyte-derived MMP-9 was increased by OGD+RO conditions compared with normal conditions (5.70-fold, *P* < 0.05) or the Ex-4 treatment alone (5.45-fold, *P* < 0.05), but Ex-4 reduced the trend in cells exposed to OGD+RO (63.48%, *P* < 0.05) (Fig. 4d). We drew the same conclusion that OGD+RO increased the secretion of VEGF-A and MMP-9 in astrocytes but ameliorated these changes after Ex-4 administration, as evidenced by single-label immunofluorescence staining (Fig. 4e and f).

**Fig. 5:**
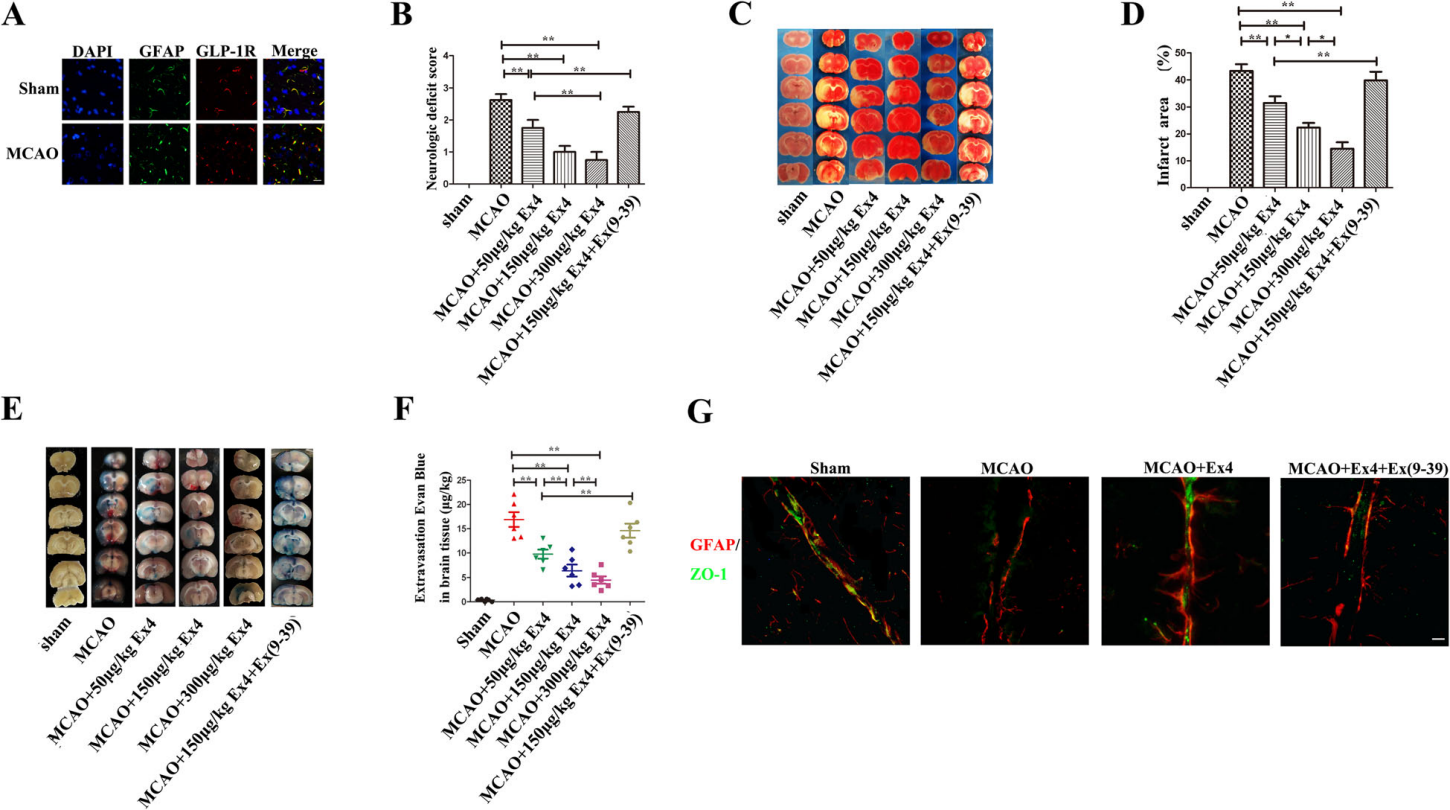
Effect of Ex-4 on neurological deficit scores, infarct area, EB invasion, and ZO-1 expression after MCAO. **a** GLP-1R (red) was expressed on GFAP (green)-positive cells in the rat brain, and the nuclei were counterstained with DAPI (blue) Scale bar, 20 μm. **b** Neurologic deficit scores of the different groups at 24 h after MCAO (*n* = 5). **c** Representative TTC staining images of brain sections from the different groups. **d** Quantification of cerebral infarct area at 24 h after MCAO. **e** Representative images of coronal brain sections from rats perfused with EB from the different groups. **f** Quantitative analysis of EB intensity in different groups by spectrofluorometry (*n* = 5). **g** Representative images of immunofluorescence staining for GFAP (red) and ZO-1 (green) in ipsilateral brain tissues (*n* = 4–5) and the concentration of Ex4 was 150 μg/kg. Scale bar, 20 μm. *P < 0.05 and **P < 0.01, ANOVA plus SNK test (**b**, **d**, **f**)

**Fig. 6:**
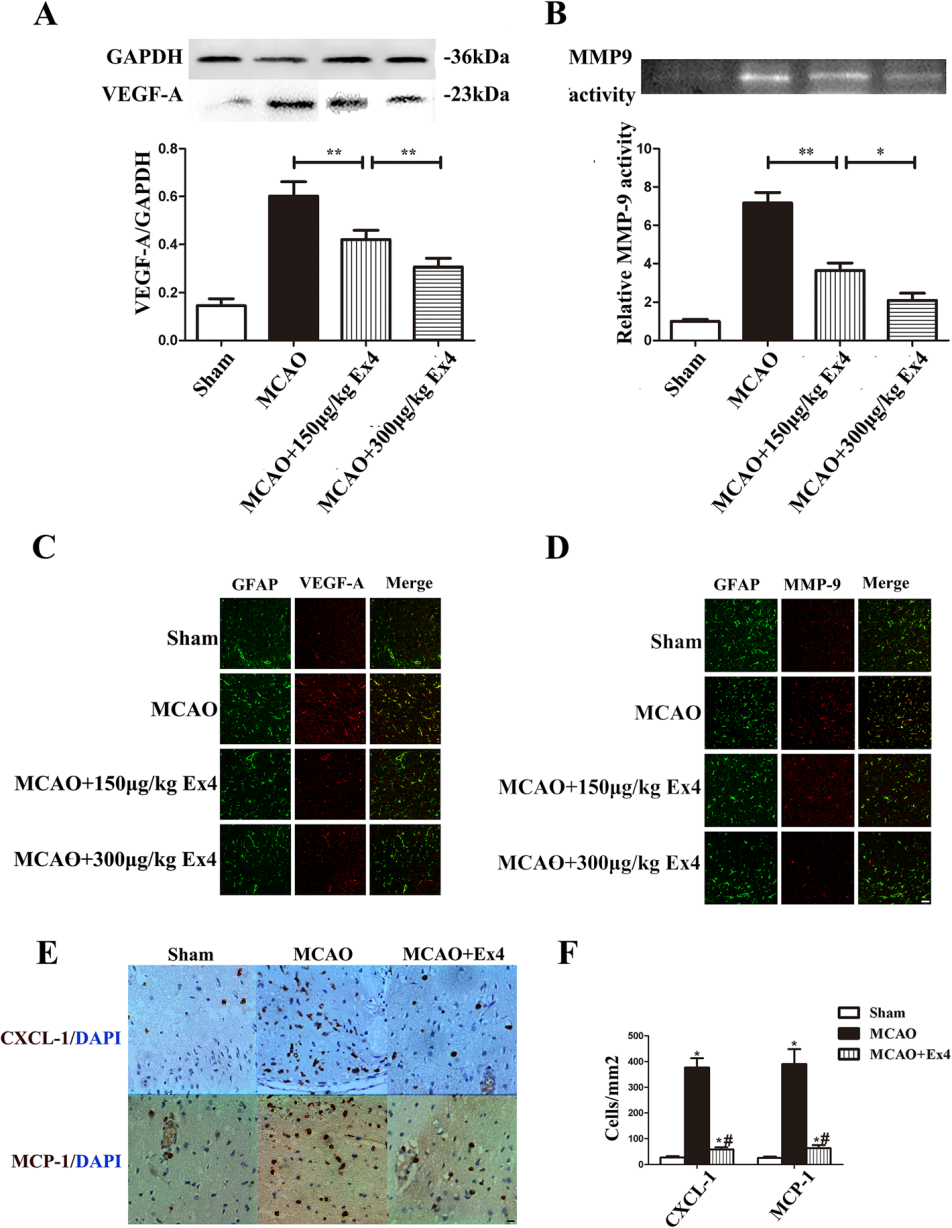
Ex-4 reduced the VEGF-A, MMP-9, CXCL-1, and MCP-1 in the infarcted brain regions of MCAO rats. **a** Representative western blots for VEGF-A expression in different models, and the VEGF-A intensity was normalized to GAPDH (*n* = 6). **b** MMP-9 activity in different models was measured using gelatin zymography, and the relative intensity was compared to the sham group (*n* = 6). **c**, **d** Representative images of immunofluorescence staining of the cerebral hemisphere from different models for VEGF-A (**c**) and MMP-9 (**d**) (red) on GFAP (green)-positive cells, and nuclei were labeled with DAPI (blue) (*n* = 5). Scale bar = 30 μm. **e** Representative images of immunohistochemistry staining for CXCL-1 and MCP-1 (brown) in ipsilateral brain tissues. **f** Quantitative analysis of CXCL-1 and MCP-1-positive cells/mm^2^ (*n* = 4). Scale bar = 20 μm. *P < 0.05 and **P < 0.01 in Fig. 6a, b; *P < 0.01 compared with the Sham group; ^#^*P* < 0.01 compared with the MCAO group in Fig. 6f; ANOVA plus SNK test (**a**, **b**, **f**)

We then confirmed the findings described above in vivo. We first detected GLP-1R expression on GFAP-positive cells in the brains of both normal and MCAO rats (Fig. 5a). The results showed that the neurologic function scores in MCAO+Ex4 group were better than those in the MCAO group and improved when the Ex-4 concentration increased (MCAO, 2.62 ± 0.18 vs. MCAO+50 μg/kg Ex4,, 1.75 ± 0.25; vs. MCAO+50 μg/kg Ex4, 0.75 ± 0.25; MCAO+50 μg/kg Ex4, 0.25 ± 0.31; all *P* < 0.01) (Fig. 5b), but the effect of Ex-4 was blocked with co-treatment with Ex(9-39). The TTC staining and quantitative analysis of EB invasion to lesions presented Ex-4 reduced the infarct area and the amount of EB in the brain of MCAO model and had the same tendency of that presented in neurologic function scores (*P* < 0.01in all MCAO+Ex4 groups vs. the MCAO group), and again, the effect was blocked by Ex(9-39) (Fig. 5c–f). As shown in Fig. 5g, immunofluorescence staining was further used to examine ZO-1 (one of the TJPs) and GFAP (representative molecules of astrocytes) to show that ZO-1 expression in the MCAO group was significantly less than that in the sham group, MCAO+Ex4 group. Moreover, ZO-1 was not tightly coexpressed with GFAP in the MCAO group; this coexpression was evident in the MCAO+Ex4 group and abolished by the administration of Ex(9-39). The results showed that Ex-4 improved the neurological deficit scores, infarct area, EB invasion, and ZO-1 expression after MCAO, and this effect was GLP-1R-dependent. Next, we further examined the effect of Ex-4 on the expression of VEGF-A (Fig. 6a and c), MMP-9 (Fig. 6b and d), CXCL-1, and MCP-1 (Fig. 6e and f) in the MCAO model. Ex-4 treatment reduced astrocyte-derived VEGF-A expression and MMP-9 in the infarcted hemispheres of MCAO rats in a dose-dependent manner, as shown by western blot analysis or gelatin zymography (Fig. 6a–d). Moreover, Ex-4 reduced the CXCL-1- and MCP-1-positive cells in the lesions of MCAO rats (Fig. 6e and f). The data in Fig. 6 were consistent with those observed with in vitro astrocyte cultures. Thus, Ex-4 reduced hypoxia-induced astrocyte-derived VEGF-A and MMP-9 levels, and the decreased CXCL-1 and MCP-1 expression in MCAO model may also be related to the effect of Ex-4 on astrocytes.

### Blockade of VEGF-A and MMP-9 in astrocytes reversed the protective effect of Ex-4 on TJPs in bEnd.3 cells

**Fig. 7:**
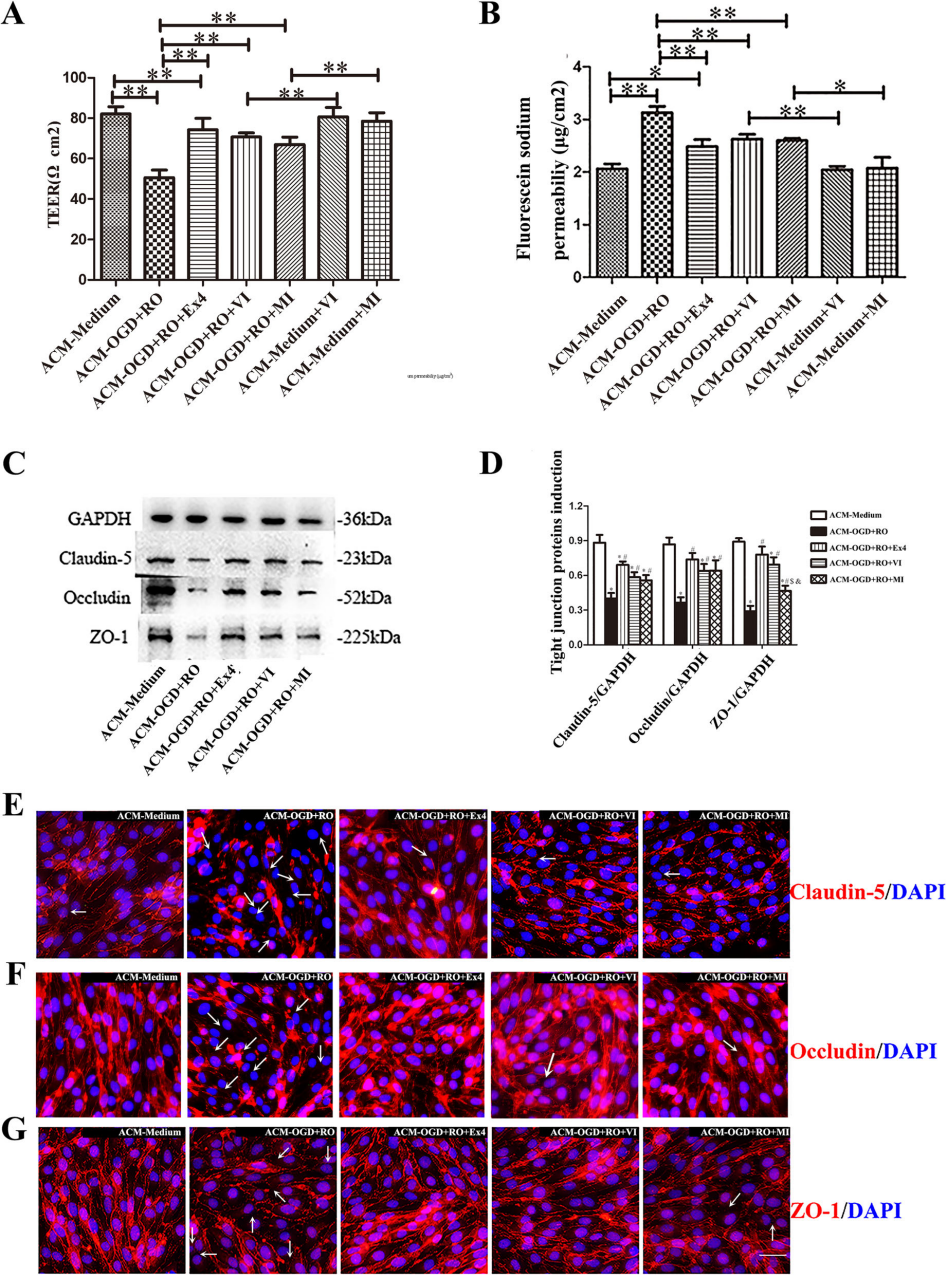
Blockade of the effects of astrocyte-derived VEGF-A and MMP-9 on the bEnd.3 monolayer protected the TJPs in bEnd.3 cells. **a** The TEER value of bEnd.3 monolayer cultured with different ACMs for 24 h was assessed. **b** The ability of NaF to cross the bEnd.3 cell monolayer cultured with different ACMs for 24 h was assessed (*n* = 6). **c** Representative western blots for TJPs, including claudin-5, occludin, and ZO-1, in bEnd.3 cells, along with the loading control GAPDH. **d** The intensity of TJPs was normalized to GAPDH. Quantitative data are presented as the mean ± SD (*n* = 6). **e**–**g** Images of immunofluorescence staining for TJPs (claudin5, occludin, and ZO-1) (red) and nuclear staining with DAPI (blue) in bEnd.3 monolayers cultured with different ACMs (*n* = 6). The arrows indicate the decreased levels of TJPs between bEnd.3 cells. Scale bar = 50 μm. ACM-Medium, cultured with ACM from untreated astrocytes; ACM-OGD+RO, cultured with ACM from astrocytes treated with OGD for 4 h plus RO for 24 h; ACM-OGD+RO+Ex-4, cultured with ACM from astrocytes treated with OGD for 4 h plus RO for 24 h in the presence of Ex-4; ACM-OGD+RO+VI, cultured with ACM from astrocytes treated with OGD for 4 h plus RO for 24 h and the anti-VEGFR2 antibody; ACM-OGD+RO+MI, cultured with ACM from astrocytes treated with OGD for 4 h plus RO for 24 h and batimastat; ACM-Medium+VI, cultured with ACM from untreated astrocytes and the anti-VEGFR2 antibody; ACM-Medium+MI, cultured with ACM from untreated astrocytes and batimastat. All bEnd.3 cell monolayers were pretreated with Ex(9-39) before culture with different ACMs. *P < 0.05 and **P < 0.01 in Fig. 7a, b; *P < 0.05 compared with the ACM-Medium group; ^#^*P* < 0.05 compared with the ACM-OGD+RO group, ^$^*P* < 0.05 compared with the ACM-OGD+RO+Ex-4 group, and ^&^*P* < 0.05 compared with the ACM-OGD+RO+VI group in Fig. 6d, ANOVA plus SNK test (**a**, **b**, **d**)

We next used an anti-VEGFR2-blocking antibody (VI) and the MMP inhibitor batimastat (MI) to confirm that VEGF-A and MMP-9 were the “bridge” between Ex-4-mediated protection of BBB integrity and astrocytes. The TEER and NaF permeability in bEnd.3 cell monolayer among ACM-Medium, ACM-Medium+VI, and ACM-Medium+MI showed no significant differences (Fig. 7a and b). In the groups cultured with ACM from astrocytes exposed to OGD+RO, the additional VI or MI treatment increased the TEER of the cell monolayer (TEER, ACM-OGD+RO 50.40 ± 3.75, vs. ACM-OGD+RO+VI 70.71 ± 2.02, vs. ACM-OGD+RO+MI 66.93 ± 3.73 Ω cm^2^, all *P* < 0.01), but the differences in TEER among the ACM-OGD+RO+Ex-4, ACM-OGD+RO+VI, and ACM-OGD+RO+MI groups were not significant (82.14 ± 4.13, 70.71 ± 2.02, and 2.60 ± 0.04 Ω·cm^2^, respectively; *P* = 0.46 and *P* = 0.77 respectively). Again, a different tendency but the same results were found with regard to NaF permeability in bEnd.3 cells (Fig. 7a and b). Then, we examined the levels of TJPs in bEnd.3 cells cultured with different ACMs using western blot analyses. Claudin-5, occludin, and ZO-1 levels of bEnd.3 cells in ACM-OGD+RO group were decreased, as expected, but TJPs levels were increased when Ex-4 or VI/MI was added to the ACM (*P* < 0.05) (Fig. 7c and d). The arrows on images of immunofluorescence staining indicated the downregulation of TJPs between bEnd.3 cells. The results were consistent with the TEER data, the permeability assay, and western blot analysis (Fig. 7e–g). Based on these data, Ex-4 protects the integrity of the BBB partially by reducing VEGF-A and MMP-9 production in astrocytes, which were increased after OGD exposure or cerebral ischemia.

### The Ex-4-induced reduction in astrocyte-derived VEGF-A levels in ACM resulted in the inactivation of the PLCγ/PKCα/eNOS pathway in endothelial cells

**Fig. 8:**
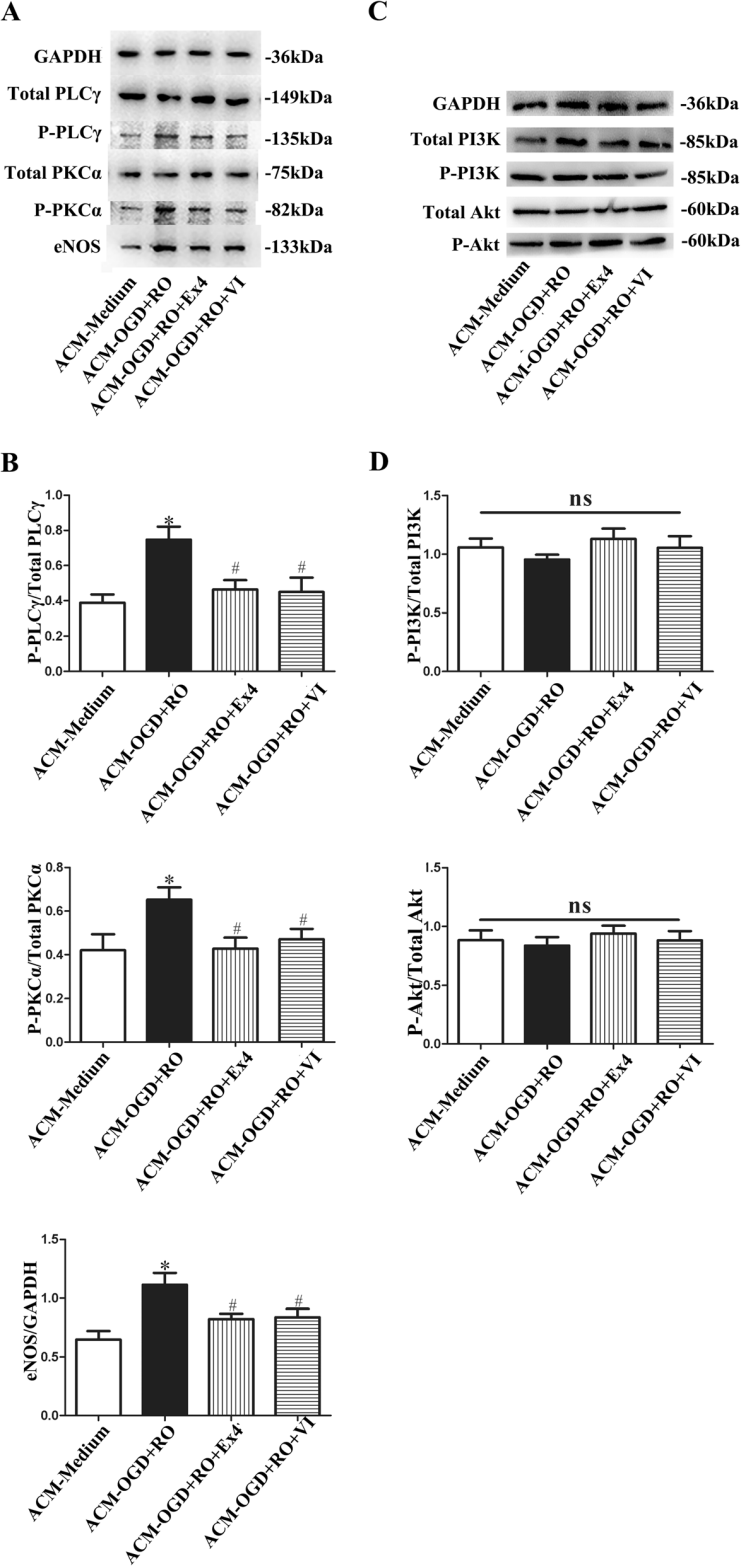
Ex-4 restored the TJPs in bEnd.3 cells by decreasing astrocyte-derived VEGF-A levels through a mechanism mediated by the PLCγ/PKCα/eNOS pathway in bEnd.3 cells. **a** Representative western blots showing the levels of p-PLCγ, p-PKCα, and eNOS in bEnd.3 cells cultured with different ACMs. Total PLCγ, total PKCα, and GADPH were used as the loading controls. **b** Quantitative analyses of p-PLCγ, p-PKCα, and eNOS levels. The data are presented as the mean ± SD (*n* = 6). **c** Representative western blots showing the levels of p-AKT and p-PI3K in bEnd.3 cells cultured with different ACMs. Total PI3K and total Akt were used as the loading controls. **d** Quantitative analyses of p-PI3K and p-Akt levels. The data are presented as the mean ± SD (*n* = 6). ACM-Medium, cultured with ACM from untreated astrocytes; ACM-OGD+RO, cultured with ACM from astrocytes treated with OGD for 4 h plus RO for 24 h; ACM-OGD+RO+Ex4, cultured with ACM from astrocytes treated with OGD for 4 h plus RO for 24 h in the presence of Ex-4; ACM-OGD+RO+VI, cultured with ACM from astrocytes treated with OGD for 4 h plus RO for 24 h and the anti-VEGFR2 antibody. All bEnd.3 cell monolayers were pretreated with Ex(9-39) before culture with different ACMs. *P < 0.05 compared with the ACM-Medium group and ^#^*P* < 0.05 compared with the ACM+OGD+RO group, ANOVA plus SNK test (**b, d**)

A previous study examining the MCAO model noted that Ex-4 preserves the BBB integrity after cerebral ischemic stroke through the PI3K/Akt pathway [17]. VEGF-A-VEGFR2 signaling activates the PLCγ/PKCα/eNOS or PI3K/Akt/eNOS pathways to disrupt TJPs in confluent endothelial cells [8]. We next investigated whether these pathways were involved in the protective effect of ACM from astrocytes treated with Ex-4 on bEnd.3 cells. Briefly, increased levels of p-PLCγ, p-PKCα, and eNOS were detected in the ACM-OGD+RO group, but the levels were decreased when Ex-4 or VI was co-cultured with the above ACM (ACM-OGD+RO, vs. ACM-Medium, vs. ACM-OGD+RO+Ex-4, vs. ACM-OGD+RO+VI, all *P* < 0.05; but ACM-OGD+RO+Ex-4 vs. ACM-OGD+RO+VI, *P* = 0.93) (Fig. 8a and b). However, the p-PI3K or p-Akt levels did not change among the different groups (Fig. 8c and d). These data revealed the mechanism by which ACM from astrocytes treated with Ex-4 decreased the permeability of endothelial cells.

### Ex-4 reduced astrocyte-derived VEGF-A expression through JAK2/STAT3 signaling

**Fig. 9:**
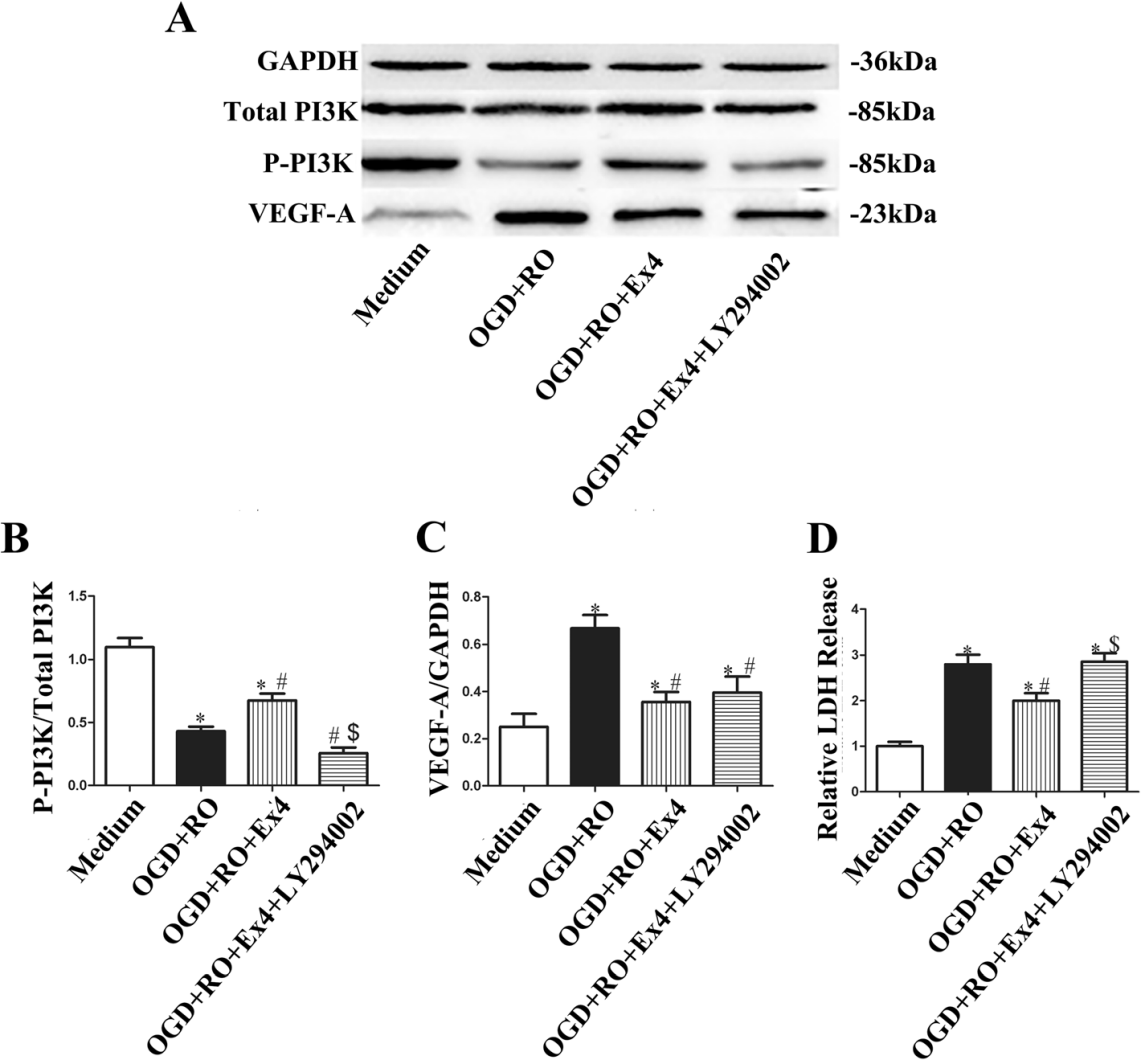
Ex-4 improved viability of astrocytes but not the secretion of VEGF-A via PI3K-related signaling. **a** Representative western blots showing the levels of VEGF-A, p-PI3K in astrocytes cultured under different conditions. Total PI3K and GADPH were used as the loading controls. **b**, **c** Quantitative analyses of VEGF-A, p-PI3K levels. The data are presented as the mean ± SD (*n* = 6). **d** LDH release of astrocytes administered different treatment was assessed. Medium, cultured with normal medium; OGD+RO, treated with OGD for 4 h plus RO for 24 h; OGD+RO+Ex4, treated with OGD for 4 h plus RO for 4 h in the presence of Ex-4, OGD+RO+Ex4+LY294002. *P < 0.05 compared with the Medium group, ^#^*P* < 0.05 compared with the OGD+RO group, ^$^*P* < 0.05 compared with the OGD+RO+Ex4 group; ANOVA plus SNK test (**b**–**d**)

**Fig. 10:**
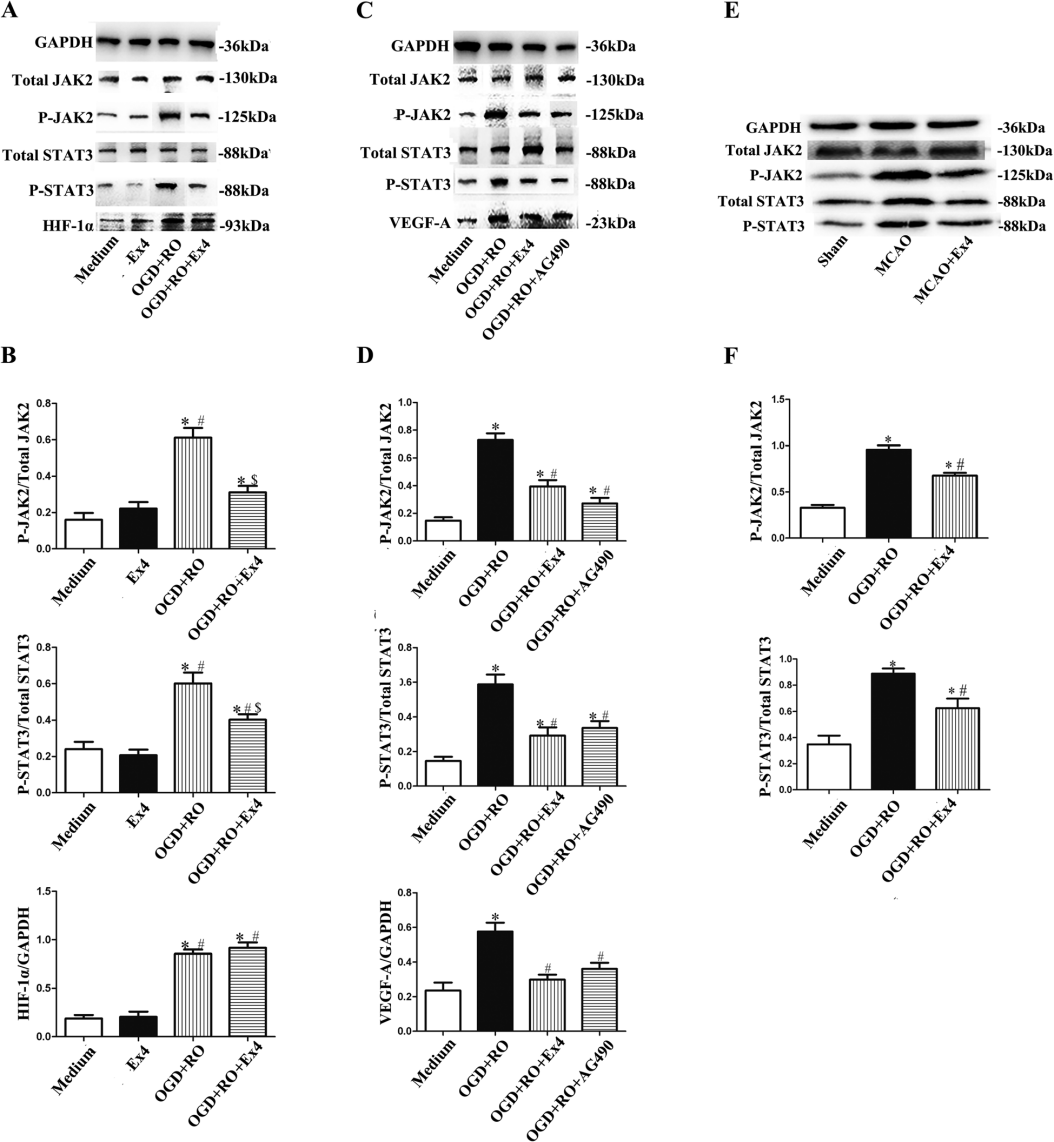
Ex-4 inhibits VEGF-A expression in astrocytes via JAK2/STAT3 signaling. **a** Representative western blots showing the levels of p-JAK2, p-STAT3, and HIF-1α in astrocytes cultured under different conditions. Total JAK2, total STAT3, and GADPH were used as the loading controls. **b** Quantitative analyses of p-JAK2, p-STAT3, and HIF-1α levels. The data are presented as the mean ± SD (*n* = 6). **c** Representative western blots showing the levels of p-JAK2, p-STAT3, and VEGF-A in astrocytes cultured under different conditions. Total JAK2, total STAT3, and GADPH were used as the loading controls. **d** Quantitative analyses of p-JAK2, p-STAT3, and VEGF levels. The data are presented as mean ± SD (*n* = 6). **e** Representative western blots for p-JAK2 and p-STAT3 expressions in different models, and total JAK2, total STAT3, and GADPH were used as the loading controls. **f** Quantitative analyses of p-JAK2 and p-STAT3 levels. The data are presented as the mean ± SD (*n* = 6). Medium, cultured with normal medium; Ex4, treated with Ex-4 for 24 h; OGD+RO, treated with OGD for 4 h plus RO for 24 h; OGD+RO+Ex4, treated with OGD for 4 h plus RO for 24 h in the presence of Ex-4; OGD+RO+AG490, treated with OGD for 4 h plus RO for 24 h with a preincubation with AG490. *P < 0.05 compared with the Medium group, ^#^*P* < 0.05 compared with the Ex4 group, ^$^*P* < 0.05 compared with the OGD+RO group in Fig. 10b; *P < 0.05 compared with the Medium group, and ^#^*P* < 0.05 compared with the OGD+RO group in Fig. 10d; *P < 0.05 compared with the Sham group, ^#^*P* < 0.05 compared with the MCAO group in Fig. 10e, ANOVA plus SNK test (**b**, **d**, **e**)

Finally, we investigated the potential mechanism by which Ex-4 reduced the OGD+RO-induced increase in VEGF-A levels in astrocytes. PI3K/Akt is the most common downstream pathway of GLP-1R activation [17]. However, although Ex-4 reduced VEGF-A, it increased PI3K phosphorylation with a decrease in LDH release in OGD-treated astrocytes (Fig. 9). The PI3K inhibitor LY294002 only blocked the effect of Ex-4 on PI3K phosphorylation and the LDH release of astrocytes, but there was no significant difference in VEGF-A production in the OGD+RO+Ex4+LY294002 group compared with the OGD+RO+Ex4 group (Fig. 9). This result indicated that the effect of Ex-4 on OGD-induced increased astrocyte-derived VEGF-A did not occur via the PI3K signaling pathway. On the other hand, HIF-1α and STAT3 have been reported to be upstream factors of VEGF-A [23]. In our study, although OGD+RO increased HIF-1α levels in astrocytes, the addition of Ex-4 did not change the expression levels of this protein (*P* = 0.78) (Fig. 10a and b). However, the levels of p-STAT3 and its upstream factor p-JAK2 in astrocytes were increased under OGD+RO conditions and reversed by Ex-4 co-treatment (OGD+RO vs. OGD+RO+Ex4 *P* < 0.05; Fig. 10a and b). The cells were then co-treated with AG490, a JAK2 inhibitor, to determine whether the activation of the JAK2/STAT3 pathway was involved in the downregulation of VEGF-A in astrocytes induced by Ex-4. Western blot results showed noticeable decreases in the levels of p-JAK2, p-STAT3, and VEGF-A in cells treated with OGD in combination with Ex-4 or AG490 (OGD+RO vs. OGD+RO+Ex4 and OGD+RO vs. OGD+RO+AG490, both *P* < 0.05; Fig. 10c and d). Besides, in the MCAO model, the levels of p-JAK2 and p-STAT3 of the infarcted hemisphere in MCAO+Ex4 group were decreased compared with those of the MCAO group (MCAO+Ex4 vs. MCAO, *P* < 0.05, Fig. 10e and f), indicating that Ex-4 downregulated the JAK2/STAT3 pathway in ischemic cerebral tissue, which was consistent with the in vitro results. Thus, the JAK2/STAT3 pathway was involved in the effect of Ex-4 on astrocytes following OGD+RO treatment through influencing the VEGF-A production and may also be related to the improvement shown in the MCAO+Ex4 group.

## Discussion

Astrocytes are the most abundant cell type within the CNS, and they provide structural support, promote the formation of the BBB, and release beneficial factors that maintain brain cell development and homeostasis of the extracellular environment [24]. The function of brain endothelial monolayer cells, ATP-binding cassette transporters, and TJP expression for example, is enhanced when the cells are co-cultured with astrocytes or ACM for a period of time [5, 25]. In the present study, ACM did not alter TJP expression or the integrity of bEnd.3 cell monolayer, potentially due to the relatively short culture period, which was consistent with the results from previous studies [11, 25]. Although astrocytes are generally more resistant to the effects of AIS than other cells [26], AIS- or OGD-induced-astrocytes aggravate BBB disruption by inflammatory factors [27], consistent with our results that ACM from astrocytes treated with OGD+RO destroyed the integrity of the bEnd.3 cell monolayer.

In the mammalian brain, GLP-1R expression has been detected in endothelial cells, neurons, astrocytes, and microglia [16, 17, 28]. The administration of a GLP-1R agonist in vitro was recently shown to strengthen the integrity of brain endothelial cells and improve cortical neuron survival under pathological conditions [16, 28] and reduce the permeability of the BBB, microglia activity, and neutrophil infiltration after MCAO in vivo [17, 29]. However, whether the effect of Ex-4 on BBB integrity (or protecting TJPs) is related to the viability of endothelial cells remains unknown because the effect is accompanied by PI3K/Akt signaling activation, a pathway related to cell viability [17]. On the other hand, although Ex-4 reduces the inflammatory factors in stroke [17], Wu et al. and Darsalia et al. found that GLP-1R activation did not inhibit LPS-induced or ischemia-induced spinal or brain expression of microglia-derived pro-inflammatory cytokines [30, 31]. Our experiment also found that Ex-4 did not reduce the inflammatory factors (IL-1β, IL-6, TNF-α) (Additional file 1: Figure S1) secreted by BV-2 cells after OGD, suggesting that Ex-4 does not directly affect the microglia secretion of inflammatory factors. The above data cannot fully explain the effect and the mechanism of Ex4 on ischemic stroke. However, researchers have not determined whether the effect of Ex-4 on AIS is related to astrocytes. The TJPs between brain endothelial cells are the physical barrier of the BBB that prevent peripheral harmful and superfluous substances from entering the brain to maintain the homeostasis of the intracerebral environment [1]. In the present study, ACM from astrocytes treated with OGD+RO destroyed the integrity of the bEnd.3 cell monolayer, but this change was reversed by Ex-4 through GLP-1R expressed on astrocytes and was not related to cell viability. Restored TJPs account for the effect of ACM from astrocytes treated with Ex-4, indicating that the components of ACM that were changed by Ex-4 ultimately affected TJPs on bEnd.3 cells to protect BBB integrity.

Astrocytes are a major source of inflammatory factors in lesions after AIS [6, 7, 10, 13, 14, 32], which contribute greatly to peripheral immune cell recruitment, microglial activation, brain damage aggravation, and BBB disruption [7, 14]. According to our data, the levels of astrocyte-secreted inflammatory factors were significantly increased by the OGD+RO treatment, consistent with the results of previous studies [6, 7, 10, 13, 14]. However, only the secretion of MCP-1, CXCL-1, VEGF-A, and MMP-9 was reduced by the Ex-4 treatment after OGD, and all 4 factors were reduced by Ex-4 treatment after MCAO. Further experiments investigating the effects of MCP-1 and CXCL-1 in an MCAO model have not performed, but it was clear that MCP-1 is involved in recruiting monocytes/macrophages and activating microglia [33, 34], and CXCL-1 is an important chemokine that recruits neutrophils to the area of the brain affected by AIS [7]. The reduction in the levels of these two chemokines in astrocytes by Ex-4 in vitro and in vivo may partially explain why Ex-4 ameliorated the microglial activation and neutrophil recruitment in previous in vivo experiments [16, 17].

Further data obtained from in vitro and in vivo experiments confirmed that Ex-4 ameliorated ischemic anoxic attack-induced astrocyte-derived VEGF-A and MMP-9 secretion. VEGF-A was found to induce vessel formation [35]. However, after AIS, VEGF-A expression is upregulated in astrocytes in the mammalian brain for up to 2 weeks [36], and VEGF-A increases BBB permeability through VEGFR2 expressed on brain endothelial cells and induces the degradation of TJPs at the beginning of AIS [9, 37]. MMP-9 levels are increased in subjects with AIS and degrade the neurovascular basal lamina or TJPs to disrupt the BBB [38, 39]. Astrocytes produce more MMPs in both the MCAO and OGD+RO models [39, 40]. Our study supported the points and illustrated that Ex-4 protected the TJPs between bEnd.3 cells via an astrocyte-dependent manner.

Ex-4 preserves the BBB integrity after MCAO by increasing PI3K/Akt signaling [17]. However, VEGF-A activates VEGFR on endothelial cells and mainly signals through the PLCγ/PKCα/eNOS or PI3K/Akt/eNOS pathways to degrade TJPs, which may contradict the results reported by Chen [17]. eNOS has been shown to participate in increasing the permeability of BBB under pathological conditions [8]. In the present study, Ex-4 ameliorated eNOS expression in bEnd.3 cells cultured with ACM from OGD-treated astrocytes, and the levels of phosphorylated PLCγ and PKCα showed the same trend, which was consistent with the effect of the eNOS inhibitor on brain endothelial cells [8]. No ACM altered the PI3K/Akt pathway in bEnd.3 cells, although PI3K/Akt is one of the downstream targets of VEGF-A-VEGFR2. This discrepancy might be attributed to the involvement of the PI3K/Akt pathway in the cell survival/death pathway through the activation of different downstream targets [41] and demonstrated that the manner of how Ex-4 improved BBB integrity through astrocytes was not related to bEnd.3 cell viability. However, brain endothelial cells are more sensitive to ischemic and anoxic attack than astrocytes [26]; thus, the direct effect of Ex-4 on brain endothelial cells may also be involved in their increased vitality.

PI3K/Akt is the most common downstream pathway of GLP-1R activation and was found to be activated by Ex-4 in many in vivo stroke models [7, 42]. However, our previous data showed that Ex-4-induced reduction in VEGF-A was not related to PI3K activation. It is known that HIF-1α plays an important role in BBB breakdown following hypoxic/ischemic injury [43]. Exposure to OGD increases HIF-1α expression in astrocytes, and one of the downstream proteins of HIF-1α is VEGF-A [23, 26]. On the other hand, our and other laboratories have found that JAK2/STAT3 or IL-6/JAK2/STAT3 activation leads to increased VEGF-A expression in different cells, including astrocytes [44, 45]. AG490, a JAK2 inhibitor, has been reported to suppress pathological processes in different diseases by inactivating the JAK2/STAT3 pathway [45, 46]. In the present study, both Ex-4 and AG490 deactivated the JAK2/STAT3 pathway in astrocytes in vitro and in vivo, accompanied by decreased levels of astrocyte-derived VEGF-A but not the levels of other pro-inflammatory cytokines and HIF-1α after OGD treatment. Based on these results, Ex-4 affected OGD+RO-treated astrocytes by decreasing the level of the phosphorylated JAK2/STAT3 protein, which is a new signaling pathway that has not previously been reported.

Our study has some limitations that should be acknowledged. First, we used only a relevant inhibitor to assess the effects of GLP-1R agonists on astrocytes in vitro and did not knock out the related genes. Similarly, GLP-1R knockout in astrocytes in vivo would make the data more convincing. Second, we only chose some familiar astrocyte-derived factors that are related to the inflammation, BBB disruption, microglia activation, and neutrophil recruitment, which may overlook some factors that are also influenced by Ex-4. Third, we focused on the mechanism by which Ex-4 treatment of astrocytes influences BBB integrity. Although OGD+RO-induced increases in the levels of astrocyte-secreted chemokines were influenced by Ex-4 treatment, we did not further confirm their possible roles in Ex-4-related microglial activation and neutrophil recruitment in vitro or in vivo. Finally, the immediate administration of Ex-4 after reperfusion through an intraperitoneal injection is not an appropriate treatment for patients in the clinic. The route of administration and the dose of this treatment require further study.

Nevertheless, our study presented evidence suggesting a vital effect of GLP-1R agonists on astrocytes in AIS. Because Ex-4 crosses the BBB, pretreatment or treatment with Ex-4 immediately after AIS may improve patient outcomes, particularly patients suffering from diabetes. Clinical trials of GLP-1R agonists and related beneficial inhibitors are needed and will focus on the safety, efficacy, and mechanism of these therapeutics in patients with AIS.

## Conclusions

GLP-1R agonist has a protective effect on ischemia by reducing inflammatory factors and BBB breakdown in an astrocyte-dependent manner. Moreover, JAK2/STAT3 signaling is involved in the effect of the GLP-1R agonist on astrocytes.

## Supplementary information

**Additional file 1: Figure S1.** Ex-4 has no effect on the inflammatory factors derived by OGD+RO-treated BV-2 cell in vitro. Levels of the IL-1β (A), IL-6 (B), TNF-α (C) proteins secreted by BV-2 cell exposed to different treatments were measured using ELISAs (*n* = 6). *P < 0.05 compared with the Medium group; #*P* < 0.05 compared with the Ex4 group, ANOVA plus SNK test (A-C).

## Data Availability

All raw data used in this manuscript are available on request.

## Abbreviations

Ex-4: Exendin-4
GLP-1R: Glucagon-like peptide-1 receptor
GFAP: Glial fibrillary acidic protein
OGD: Oxygen-glucose deprivation
RO: Reoxygenation
BBB: Blood-brain barrier
TJP: Tight junction protein
ACM: Astrocyte-conditioned medium
MCP-1: Chemokines monocyte chemoattractant protein-1
CXCL-1: Chemokine C-X-C motif ligand 1
VEGF-A: Vascular endothelial growth factor
MMP: Matrix metalloproteinase
MCAO: Middle cerebral artery occlusion
JAK2: Janus kinase 2
PLCγ: Phospholipase Cγ

## References

[1] Yang Y, Rosenberg G (2011). Blood-brain barrier breakdown in acute and chronic cerebrovascular disease. Stroke.

[2] Sá-Pereira I, Brites D, Brito M (2012). Neurovascular unit: a focus on pericytes. Mol Neurobiol.

[3] Willis C, Leach L, Clarke G, Nolan C, Ray D (2004). Reversible disruption of tight junction complexes in the rat blood-brain barrier, following transitory focal astrocyte loss. Glia.

[4] Shin J, Kim Y, Jeong S, Lee K, Kim H, Park E (2015). Extracellular signal-regulated kinase1/2-dependent changes in tight junctions after ischemic preconditioning contributes to tolerance induction after ischemic stroke. Brain Struct Funct.

[5] Nakagawa S, Deli MA, Kawaguchi H, Shimizudani T, Shimono T, Kittel A, Tanaka K, Niwa M (2009). A new blood-brain barrier model using primary rat brain endothelial cells, pericytes and astrocytes. Neurochem Int.

[6] Wiese S, Karus M, Faissner A (2012). Astrocytes as a source for extracellular matrix molecules and cytokines. Front Pharmacol.

[7] Gelderblom M, Weymar A, Bernreuther C, Velden J, Arunachalam P, Steinbach K, Orthey E, Arumugam TV, Leypoldt F, Simova O (2012). Neutralization of the IL-17 axis diminishes neutrophil invasion and protects from ischemic stroke. Blood.

[8] Argaw A, Asp L, Zhang J, Navrazhina K, Pham T, Mariani J, Mahase S, Dutta D, Seto J, Kramer E (2012). Astrocyte-derived VEGF-A drives blood-brain barrier disruption in CNS inflammatory disease. J Clin Invest.

[9] Zhang Z, Zhang L, Jiang Q, Zhang R, Davies K, Powers C, Bruggen N, Chopp M (2000). VEGF enhances angiogenesis and promotes blood-brain barrier leakage in the ischemic brain. J Clin Invest.

[10] Ceruti S, Colombo L, Magni G, Viganò F, Boccazzi M, Deli MA, Sperlágh B, Abbracchio MP, Kittel A (2011). Oxygen-glucose deprivation increases the enzymatic activity and the microvesicle-mediated release of ectonucleotidases in the cells composing the blood-brain barrier. Neurochem Int.

[11] Neuhaus W, Gaiser F, Mahringer A, Franz J, Riethmüller C, Fcörster C (2014). The pivotal role of astrocytes in an in vitro stroke model of the blood-brain barrier. Front Cell Neurosci.

[12] Wang S, Lee SR, Guo SZ, Kim WJ, Montaner J, Wang X, Lo EH (2006). Reduction of tissue plasminogen activator-induced matrix metalloproteinase-9 by simvastatin in astrocytes. Stroke.

[13] Chen Y, Hallenbeck JM, Ruetzler C, Bol D, Thomas K, Berman NE, Vogel SN (2003). Overexpression of monocyte chemoattractant protein 1 in the brain exacerbates ischemic brain injury and is associated with recruitment of inflammatory cells. J Cereb Blood Flow Metab.

[14] Strecker JK, Minnerup J, Gess B, Ringelstein EB, Schäbitz WR, Schilling M (2011). Monocyte chemoattractant protein-1-deficiency impairs the expression of IL-6, IL-1β and G-CSF after transient focal ischemia in mice. PLoS One.

[15] Hakon J, Ruscher K, Romner B, Tomasevic G (2015). Preservation of the blood brain barrier and cortical neuronal tissue by liraglutide, a long acting glucagon-like-1 analogue, after experimental traumatic brain injury. PLoS One.

[16] Fukuda S, Nakagawa S, Tatsumi R, Morofuji Y, Takeshita T, Hayashi K, Tanaka K, Matsuo T, Niwa M (2016). Glucagon-like peptide-1 strengthens the barrier integrity in primary cultures of rat brain endothelial cells under basal and hyperglycemia conditions. J Mol Neurosci.

[17] Chen F, Wang W, Ding H, Yang Q, Dong Q, Cui M (2016). The glucagon-like peptide-1 receptor agonist exendin-4 ameliorates warfarin-associated hemorrhagic transformation after cerebral ischemia. J Neuroinflammation.

[18] Reiner D, Mietlicki-Baase E, McGrath L, Zimmer D, Bence K, Sousa G, Konanur V, Krawczyk J, Burk D, Kanoski S (2016). Astrocytes regulate GLP-1 receptor-mediated effects on energy balance. J Neurosci.

[19] Rubin LL, Hall DE, Porter S, Barbu K, Cannon C, Horner HC, Janatpour M, Liaw CW, Manning K, Morales J (1991). A cell culture model of the blood-brain barrier. J Cell Biol.

[20] Baez-Jurado E, Hidalgo-Lanussa O, Guio-Vega G, Ashraf GM, Echeverria V, Aliev G, Barreto GE (2018). Conditioned medium of human adipose mesenchymal stem cells increases wound closure and protects human astrocytes following scratch assay in vitro. Mol Neurobiol.

[21] Longa EZ, Weinstein PR, Carlson S, Cummins R (1989). Reversible middle cerebral artery occlusion without craniectomy in rats. Stroke.

[22] Zhang L, Huang Y, Lin Y, Shan Y, Tan S, Cai W, Li H, Zhang B, Men X, Lu Z (2016). Anti-inflammatory effect of cholera toxin B subunit in experimental stroke. J Neuroinflammation.

[23] Schoch H, Fischer S, Marti H (2002). Hypoxia-induced vascular endothelial growth factor expression causes vascular leakage in the brain. Brain.

[24] Ransom B, Ransom C (2012). Astrocytes: multitalented stars of the central nervous system. Methods Mol Biol.

[25] Puech C, Hodin S, Forest V, He Z, Mismetti P, Delavenne X, Perek N (2018). Assessment of HBEC-5i endothelial cell line cultivated in astrocyte conditioned medium as a human blood-brain barrier model for ABC drug transport studies. Int J Pharm.

[26] Engelhardt S, Huang S, Patkar S, Gassmann M, Ogunshola O (2015). Differential responses of blood-brain barrier associated cells to hypoxia and ischemia: a comparative study. Fluids Barriers CNS.

[27] Liu Z, Chopp M (2016). Astrocytes, therapeutic targets for neuroprotection and neurorestoration in ischemic stroke. Prog Neurobiol.

[28] Wang M, Huang Y, Zhang G, Mao L, Xia Y, Mei Y, Hu B (2012). Exendin-4 improved rat cortical neuron survival under oxygen/glucose deprivation through PKA pathway. Neuroscience.

[29] Hou J, Manaenko A, Hakon J, Hansen-Schwartz J, Tang J, Zhang JH (2012). Liraglutide, a long-acting GLP-1 mimetic, and its metabolite attenuate inflammation after intracerebral hemorrhage. J Cereb Blood Flow Metab.

[30] Wu HY, Tang XQ, Mao XF, Wang YX (2017). Autocrine interleukin-10 mediates glucagon-like peptide-1 receptor-induced spinal microglial β-endorphin expression. J Neurosci.

[31] Darsalia V, Hua S, Larsson M (2014). Exendin-4 reduces ischemic brain injury in normal and aged type 2 diabetic mice and promotes microglial M2 polarization. PLoS One.

[32] Chen C, Chu SF, Liu DD, Zhang Z, Kong LL, Zhou X, Chen NH (2018). Chemokines play complex roles in cerebral ischemia. Neurochem Int.

[33] Koyama Y, Kotani M, Sawamura T, Kuribayashi M, Konishi R, Michinaga S (2013). Different actions of endothelin-1 on chemokine production in rat cultured astrocytes: reduction of CX3CL1/fractalkine and an increase in CCL2/MCP-1 and CXCL1/CINC-1. J Neuroinflammation.

[34] He M, Dong H, Huang Y, Lu S, Zhang S, Qian Y, Jin W (2016). Astrocyte-derived CCL2 is associated with M1 activation and recruitment of cultured microglial cells. Cell Physiol Biochem.

[35] Carmeliet P, Ferreira V, Breier G, Pollefeyt S, Kieckens L, Gertsenstein M, Fahrig M, Vandenhoeck A, Harpal K, Eberhardt C (1996). Abnormal blood vessel development and lethality in embryos lacking a single VEGF allele. Nature.

[36] Mărgăritescu O, Pirici D, Mărgăritescu C (2011). VEGF expression in human brain tissue after acute ischemic stroke. Rom J Morphol Embryol.

[37] Li Y, Pan R, Qin X, Yang W, Qi Z, Liu W, Liu K (2014). Ischemic neurons activate astrocytes to disrupt endothelial barrier via increasing VEGF expression. J Neurochem.

[38] Asahi M, Wang X, Mori T, Sumii T, Jung J, Moskowitz M, Fini M, Lo E (2001). Effects of matrix metalloproteinase-9 gene knock-out on the proteolysis of blood-brain barrier and white matter components after cerebral ischemia. J Neurosci.

[39] Ou-Yang L, Liu Y, Wang B, Cao P, Zhang J, Huang Y, Shen Y, Lyu J (2018). Carnosine suppresses oxygen-glucose deprivation/recovery-induced proliferation and migration of reactive astrocytes of rats in vitro. Acta Pharmacol Sin.

[40] Tejima E, Zhao BQ, Tsuji K, Rosell A, van Leyen K, Gonzalez RG, Montaner J, Wang X, Lo EH (2007). Astrocytic induction of matrix metalloproteinase-9 and edema in brain hemorrhage. J Cereb Blood Flow Metab.

[41] Cross D, Alessi D, Cohen P, Andjelkovich M, Hemmings B (1995). Inhibition of glycogen synthase kinase-3 by insulin mediated by protein kinase B. Nature.

[42] Cho YM, Fujita Y, Kieffer TJ (2014). Glucagon-like peptide-1: glucose homeostasis and beyond. Annu Rev Physiol.

[43] Ogunshola O, Al-Ahmad A (2012). HIF-1 at the blood-brain barrier: a mediator of permeability?. High Alt Med Biol.

[44] Mitsuyama K, Matsumoto S, Masuda J, Yamasakii H, Kuwaki K, Takedatsu H, Sata M (2007). Therapeutic strategies for targeting the IL-6/STAT3 cytokine signaling pathway in inflammatory bowel disease. Anticancer Res.

[45] Deng Z, Wang Y, Zhou L, Shan Y, Tan S, Cai W, Liao S, Peng L, Lu Z (2017). High salt-induced activation and expression of inflammatory cytokines in cultured astrocytes. Cell Cycle.

[46] Park J, Lee J, Lim M, Kim E, Kim S, Ryu J, Lee J, Kwok S, Park K, Kim H (2014). JAK2-STAT3 blockade by AG490 suppresses autoimmune arthritis in mice via reciprocal regulation of regulatory T Cells and Th17 cells. J Immunol.

