# Supplementary figures and images for: The glucagon-like peptide-1 receptor agonist reduces inflammation and blood-brain barrier breakdown in an astrocyte-dependent manner in experimental stroke
Source: J Neuroinflammation. 2019 Nov 28;16:242. doi: 10.1186/s12974-019-1638-6 (PMC6883580; doi:10.1186/s12974-019-1638-6)

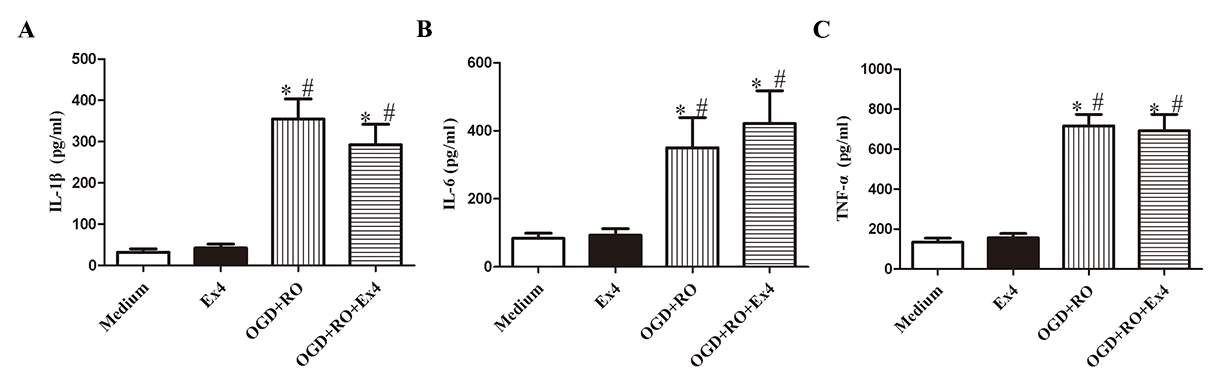

Supplement: Supplementary file 1 — Additional file 1: Figure S1. Ex-4 has no effect on the inflammatory factors derived by OGD+RO-treated BV-2 cell in vitro. Levels of the IL-1β (A), IL-6 (B), TNF-α (C) proteins secreted by BV-2 cell exposed to different treatments were measured using ELISAs (n = 6). *P < 0.05 compared with the Medium group; #P < 0.05 compared with the Ex4 group, ANOVA plus SNK test (A-C). [file 12974_2019_1638_MOESM1_ESM.tif]
